# Seabed Resuspension in the Chesapeake Bay: Implications for Biogeochemical Cycling and Hypoxia

**DOI:** 10.1007/s12237-020-00763-8

**Published:** 2020-06-09

**Authors:** Julia M. Moriarty, Marjorie A. M. Friedrichs, Courtney K. Harris

**Affiliations:** 1 Virginia Institute of Marine Science, William & Mary, Gloucester Point, VA 23062 USA; 2 Institute of Arctic and Alpine Research and Department of Atmospheric and Oceanic Sciences, University of Colorado Boulder, Boulder, CO 80303 USA

**Keywords:** Chesapeake Bay numerical model, Sediment transport, Primary production, Remineralization, Biogeochemistry, Hypoxia

## Abstract

Sediment processes, including resuspension and transport, affect water quality in estuaries by altering light attenuation, primary productivity, and organic matter remineralization, which then influence oxygen and nitrogen dynamics. The relative importance of these processes on oxygen and nitrogen dynamics varies in space and time due to multiple factors and is difficult to measure, however, motivating a modeling approach to quantify how sediment resuspension and transport affect estuarine biogeochemistry. Results from a coupled hydrodynamic–sediment transport–biogeochemical model of the Chesapeake Bay for the summers of 2002 and 2003 showed that resuspension increased light attenuation, especially in the northernmost portion of the Bay, shifting primary production downstream. Resuspension also increased remineralization in the central Bay, which experienced larger organic matter concentrations due to the downstream shift in primary productivity and estuarine circulation. As a result, oxygen decreased and ammonium increased throughout the Bay in the bottom portion of the water column, due to reduced photosynthesis in the northernmost portion of the Bay and increased remineralization in the central Bay. Averaged over the channel, resuspension decreased oxygen by ~ 25% and increased ammonium by ~ 50% for the bottom water column. Changes due to resuspension were of the same order of magnitude as, and generally exceeded, short-term variations within individual summers, as well as interannual variability between 2002 and 2003, which were wet and dry years, respectively. Our results quantify the degree to which sediment resuspension and transport affect biogeochemistry, and provide insight into how coastal systems may respond to management efforts and environmental changes.

**Electronic supplementary material:**

The online version of this article (10.1007/s12237-020-00763-8) contains supplementary material, which is available to authorized users.

## Introduction

Seabed resuspension has been observed to affect water column biogeochemistry, but its effects have been difficult to quantify in coastal systems, which typically exhibit high spatial and temporal variability (McKee et al. 2004). Resuspension entrains inorganic particulates and particulate organic matter (POM) into the water column, increasing turbidity and light attenuation (e.g., Cloern 1987; Xu et al. 2005; Gallegos et al. 2011; Shi et al. 2013). Transference of material from the seabed to the water column may also enhance remineralization rates due to the increased POM concentrations in bottom waters, as well as the exposure of that organic matter to an oxic water column (Stahlberg et al. 2006; Aller 1998; Hartnett et al. 1998; Burdige 2007; Queste et al. 2016; Bianucci et al. 2018). Resuspension may also influence fluxes of dissolved oxygen and nutrients through the seabed–water interface (e.g., Glud 2008; Toussaint et al. 2014). Additional observational studies indicate that once particulates and porewater are entrained into the water column, they may be redistributed via advection, altering spatial and temporal gradients in biogeochemical processes (e.g., Lampitt et al. 1995; Christiansen et al. 1997; Abril et al. 1999; Goñi et al. 2007). Field and laboratory approaches typically have limited spatial and/or temporal coverage, however, complicating efforts to quantify the impact of resuspension on estuarine biogeochemistry in dynamic coastal systems.

Recent numerical modeling developments have made investigations into the impact of resuspension on water column biogeochemistry feasible. Open-source hydrodynamic models have been coupled to both sediment transport (e.g., Warner et al. 2008) and water column biogeochemistry models (e.g., Fennel et al. 2006). Studies have also begun to link biogeochemistry with some subsets of sediment processes in coupled models (e.g., Testa et al. 2014; Feng et al. 2015; McSweeney et al. 2016; Capet et al. 2016; Lajaunie-Salla et al. 2017; Lu et al. 2018). Our recently developed HydroBioSed model is unique in that it directly couples process models of both sediment transport and biogeochemistry within a hydrodynamic model in order to account for the effect of resuspension on both remineralization and seabed–water column fluxes (Moriarty et al. 2017, 2018), as well as light attenuation (this study). Past implementations of HydroBioSed focused on the Rhône River subaqueous delta (Moriarty et al. 2017) and the northern Gulf of Mexico shelf (Moriarty et al. 2018) and targeted near-seabed processes on continental shelves, neglecting resuspension-induced effects on light attenuation. In contrast, this paper modifies HydroBioSed for application to an estuary, Chesapeake Bay, and focuses on quantifying the role of resuspension on both light attenuation and remineralization, as well as their impact on oxygen and nitrogen dynamics in the Bay.

**Fig. 1:**
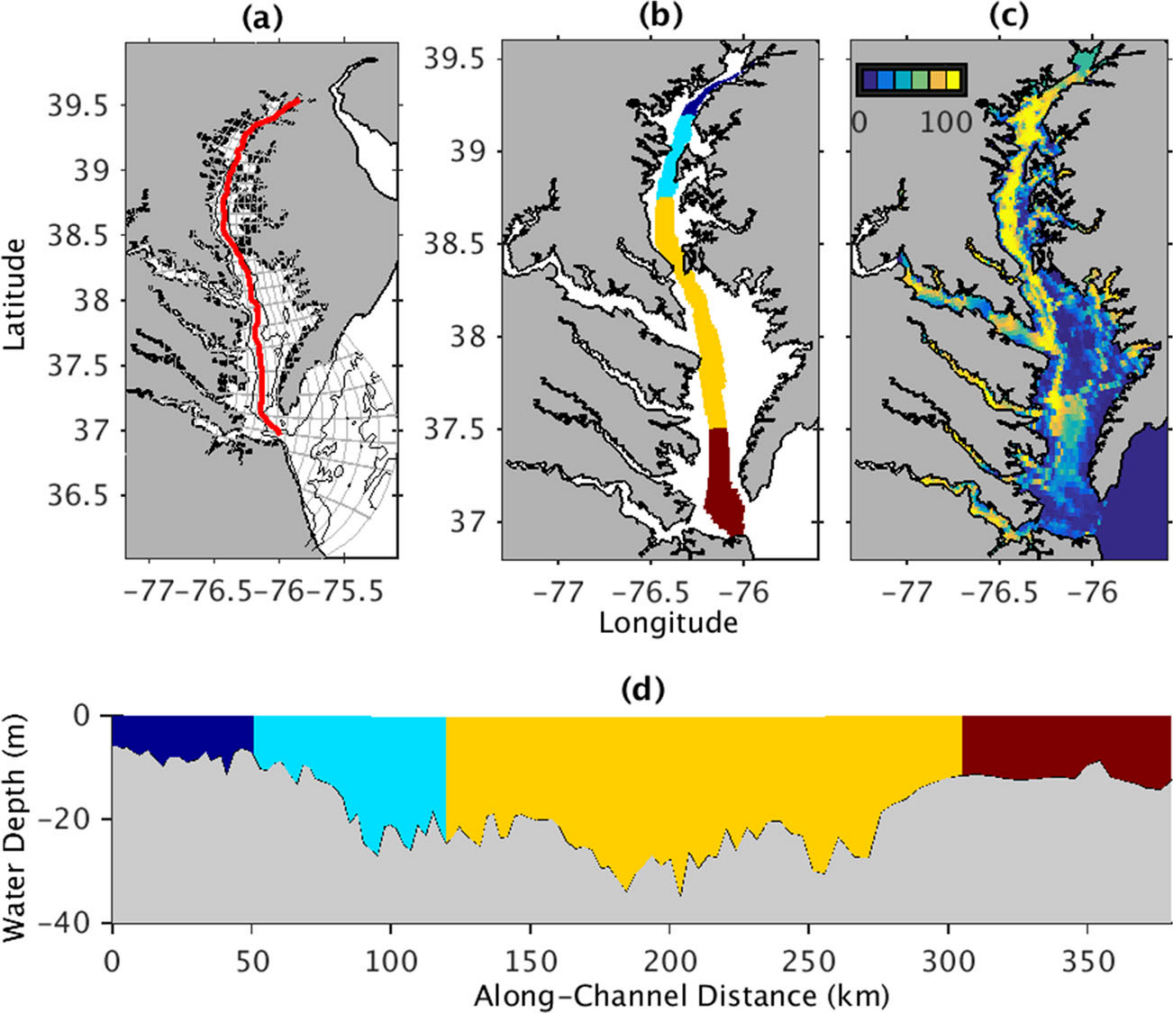
Study site maps showing the **a** model grid **b**, **d** different spatial regions considered in this study, and **c** percent of the initial seabed that is mud. In **a**, the gray boxes indicate every 25 grid cells, black lines are bathymetric contours for every 10 m, and the red line indicates the location of the along-estuary transects for **c** and Figs. 3 and 5. In **b** and **d**, each color indicates a different region of the thalweg used in the analysis, including the Oligohaline Bay (dark blue; 39.21–39.53° N), Upper Bay (turquoise; 38.77–39.21° N), Mid Bay (yellow; 37.53–38.77° N), and Lower Bay (dark red; 36.98–37.53° N). The regions include model grid cells that were deeper than 5 m. Water depths for locations along the transect are shown in **d**

The Chesapeake Bay, the largest estuary in the continental United States, receives seasonally varying inputs of freshwater, sediment, and nutrients and is characterized by a deep channel and broad shoals (Fig. 1). Phytoplankton growth depends on both nutrients and light availability. The northernmost portion of the Bay is typically considered primarily light-limited, whereas phytoplankton growth in the remainder of the Bay is generally nutrient-limited, despite eutrophication of the estuary over the past several decades (Harding et al. 2002 and references therein). As a result, the springtime delivery of nutrients stimulates primary productivity by phytoplankton in this region (e.g., Malone et al. 1996; Harding et al. 2002 and references therein). Seasonal enhancement in production and eventual decomposition of organic matter causes low oxygen levels and high ammonium concentrations to occur in the channel of the Chesapeake Bay during summer months (Kemp et al. 2005). In contrast, the shallower shoals are generally vertically mixed, and so hypoxia, i.e., the occurrence of oxygen concentrations below 2 mg L^−1^, is typically constrained to the deeper main channel. The volume of this low oxygen “dead zone” varies depending on stratification and circulation, e.g., due to wind, as well as changes in oxygen consumption, e.g., due to nutrient and organic matter availability (Scully 2010; Murphy et al. 2011; Testa and Kemp 2012).

Particulate dynamics in the Chesapeake Bay are influenced by riverine discharge, resuspension, and the formation of an estuarine turbidity maximum (ETM). The predominant feature of spatial variability in surface water total suspended solids (TSS) is the ETM, where concentrations typically peak at ~ 30–50 mg L^−1^ (Sanford et al. 2001; Son and Wang 2012; Cerco et al. 2013). Observations show that resuspension helps maintain the ETM, which is generally located between 39.2° and 39.5° N latitude, but shifts up and down the estuary depending on riverine discharge (Sanford et al. 2001). Resuspension can be induced by tides, currents, and waves (Sanford 1994; Harris et al. 2012). Higher rates of resuspension on the shoals compared to the deeper channel, combined with estuarine circulation patterns, cause particulates to accumulate in the channel (Hobbs et al. 1992; Sanford 1994; Cerco et al. 2013). Additionally, sediment accumulates in the channel due to convergence of down-thalweg fluxes north of the ETM and up-thalweg sediment fluxes in the southern Bay (Hobbs et al. 1992).

Observations indicate that seabed and sediment processes affect the biogeochemistry of the Chesapeake Bay and its tributaries. For example, in the York River estuary, a tributary of the Chesapeake Bay, observations show that resuspension enhances remineralization rates and reduces the rate of accumulation of organic matter in the seabed on timescales of years to decades (Arzayus and Canuel 2004). In Chesapeake Bay, eutrophication-induced seabed accumulation of organic matter has been linked to an observed increase in ammonium levels and hypoxic volume over the last few decades (Testa and Kemp 2012; Hagy et al. 2004; Bever et al. 2013). On daily to seasonal timescales, water column turbidity limits primary productivity, especially in the northernmost portion of the Bay (Harding et al. 2002). Together, these Chesapeake Bay studies indicate that sediment processes in general, and resuspension in particular, may affect remineralization rates and phytoplankton growth, as well as nutrient and oxygen levels. However, none of these studies could directly quantify the impact of resuspension, on biogeochemistry, or how it varied in time or space.

Sediment processes, including resuspension, have also been suggested to explain differences between biogeochemical observations and model results within the Chesapeake Bay. For example, Cerco et al. (2013) suggested that transport of POM from the shoals to the channel may help explain why their model overestimated oxygen concentrations in the channel. Xu and Hood (2006) similarly suggested that underestimating this lateral transport, or light attenuation due to resuspended sediments, accounted for their overestimation of chlorophyll on the estuary’s shoals. Finally, Li et al. (2015) indicated that changes in primary productivity by phytoplankton, e.g., via light attenuation, had a large effect on the volume of hypoxic water that developed in their model. Overall, these modeling studies suggest that Chesapeake Bay water column biogeochemistry is sensitive to seabed and sediment processes, but none of these studies have attempted to quantify the impact of resuspension on oxygen or nitrogen dynamics.

This uncertainty about the extent of resuspension’s impact on water column biogeochemistry, especially oxygen and nitrogen dynamics, motivated the implementation of our coupled hydrodynamic–sediment transport–biogeochemical model for the Chesapeake Bay. Specifically, using this model allowed us to isolate and quantify the role of resuspension and subsequent particulate transport on light attenuation, primary productivity, and remineralization, as well as to analyze how the resulting changes in these biogeochemical processes influence concentrations of oxygen and ammonium during summer months. Analysis of the model results specifically focused on (1) variations along the Chesapeake Bay estuary, particularly in the main channel where hypoxia is most problematic, and (2) interannual variability during years with high versus low river input.

## Methods

### HydroBioSed Formulations

Model formulations were added to a previous version of HydroBioSed, the coupled hydrodynamic–sediment transport–biogeochemical model developed and described by Moriarty et al. (2017, 2018). HydroBioSed was developed within the Regional Ocean Modeling System (ROMS) framework (Shchepetkin and McWilliams 2005), which incorporates the Community Sediment Transport Modeling System (CSTMS) (Warner et al. 2008), water column biogeochemistry models (e.g., Fennel et al. 2006; Feng et al. 2015), and the Soetaert et al. (1996a, 1996b) seabed diagenetic model. Consistent with previous versions of HydroBioSed, the model formulations account for processes including the transport of water, sediment, and biogeochemical tracers; the sinking and deposition of sediment and POM to the seabed; subsequent resuspension or storage of sediment and POM in the seabed; remineralization of organic matter and oxidation of reduced chemical species in both the water column and seabed; and diffusion of dissolved chemical species across the seabed–water interface. For this study, we also added formulations so that the suspended sediment and POM affect light attenuation in the model, as described at the end of this section.

HydroBioSed’s equations for erosion and deposition follow Warner et al. (2008) and were detailed in Moriarty et al. (2017, 2018), but are summarized here because this study focuses on resuspension. The model accounts for multiple sediment classes, and net fluxes of particulates across the seabed–water interface are estimated as the difference between erosion and deposition, which occur simultaneously. For sediment in class *ised* in grid cell (*i,j*), the rate of erosion, *E*_*ised,i,j*_, is calculated as follows:

1 $$ {E}_{ised,i,j}=\left\{\begin{array}{cc}M\left(1-\phi \right){f}_{ised,i,j}\left(\frac{\tau_{\mathrm{bed},i,j}-{\tau}_{\mathrm{crit}, ised,i,j}}{\tau_{\mathrm{crit}, ised,i,j}}\right)& \mathrm{if}{\tau}_{\mathrm{bed},i,j}\ge {\tau}_{\mathrm{crit}, ised,i,j}\\ {}0& \mathrm{if}{\tau}_{\mathrm{bed},i,j}<{\tau}_{\mathrm{crit}, ised,i,j}\end{array}\right\} $$

Parameters include the combined wave-plus-current-induced bed shear stress, *τ*_bed,*i,j*_; the critical stress of erosion for the sediment class, *τ*_crit,*ised*,*i,j*_; an erosion rate parameter, *M*; the fraction of the seabed composed of the individual sediment class, *f*_*ised*,*i,j*_; and the seabed porosity, *ϕ*. Erosion may therefore occur in the model when and where *τ*_bed,*i,j*_ exceeds *τ*_crit,*ised*,*i,j*_, and the erosion rate varies depending on hydrodynamic conditions and the local seabed grain size distribution. Deposition on the seabed is calculated as the product of suspended sediment concentration and particle settling velocity. These parameterizations enable the model to account for variations in erosion due to spatial and temporal changes in the seabed sediment distribution, seabed armoring, and hydrodynamic conditions. As in previous versions of HydroBioSed, POM is deposited in the same manner as inorganic particles and is eroded with the sediment classes representing mud. Once eroded into the water column, particle transport depends on the hydrodynamic conditions and particle settling velocities. Variations in transport, as well as in seabed erosion and deposition, cause water column and seabed sediment distributions to vary in space and time.

To represent water column biogeochemistry, HydroBioSed previously incorporated the Fennel et al. (2006, 2013) model (Moriarty et al. 2017, 2018), but in this study, the estuarine–carbon–biogeochemistry (ECB; Feng et al. 2015) model was used instead. The framework of ECB is similar to Fennel et al. (2006, 2013), but it includes the dissolved organic matter cycling of Druon et al. (2010) and was specifically formulated for estuaries. Consistent with previous versions of HydroBioSed, this water column model is nitrogen-based, and estimates of particulate organic carbon (POC), primary production, and remineralization in carbon-based units were estimated using nitrogen to carbon ratios (Table 1). ECB has previously been implemented within ROMS for the Chesapeake Bay (e.g., Feng et al. 2015; Irby and Friedrichs 2019; Irby et al. 2018; Da et al. 2018). Specifically, the version from Irby and Friedrichs (2019) was adapted for use in HydroBioSed. Unlike previous implementations of ECB, which incorporated simpler parameterizations of resuspension and seabed biogeochemical processes, HydroBioSed relies on its more process-based seabed biogeochemistry and sediment transport model equations to calculate resuspension of both inorganic and organic particulates (as described above), as well as fluxes of dissolved oxygen and nitrogen species between the seabed and the water column (see Moriarty et al. 2017). HydroBioSed, for example, accounts for spatially and temporally varying erodibility, as described above, as well as spatial and temporal variations in grain size in the water column and seabed. HydroBioSed also treats seabed organic matter particles as a sediment class that could later be re-entrained into the water column. In contrast, previous versions of ECB (e.g., Feng et al. 2015) accounted for one class of inorganic sediment within the water column and parameterized resuspension of organic particulates by assuming that a fraction of the POM settling to the seabed was instantaneously resuspended as small detritus, depending on the estimated bed stress. The remaining fraction of POM reaching the seabed in the previous implementations was either instantaneously remineralized or permanently buried and could not be resuspended back into the water column (Feng et al. 2015).

**Table 1:** Selected model parameters for the Reference model run

Parameter		Modeled value	Source for observed/literature values
Sediment transport parameters			
Partitioning of sediment into classes		Unaggregated mud—4 mg L^−1^ Small flocs, large flocs, and sand-ranges based on estimates from EPA’s watershed model	Cerco et al. (2010, 2013)
Settling velocity		Unaggregated mud—0.012 mm s^−1^ Small flocs—0.03 mm s^−1^ Large flocs—0.1 mm s^−1^ Sand—1.0 mm s^−1^	Cerco et al. (2010, 2013)
Median sediment grain diameter		Unaggregated mud—0.003 mm Small flocs—0.003 mm Large flocs—0.03 mm Sand—0.3 mm	Cerco et al. (2010)
Critical bed shear stress for erosion		Unaggregated mud—0.03 Pa Small flocs—0.03 Pa Large flocs—0.03 Pa Sand—20.0 Pa	Mud: Cerco et al. (2010, 2013). Sand: value chosen to match EPA (2012) data
Erosion rate parameter		3 × 10^−5^ kg m^−2^ s^−1^	Chosen to match EPA (2012) data
Porosity		0.9	Dellapenna et al. (2003)
Seabed initialization for different sediment classes		Spatially variable, based on maps of observed grain size; see Fig. 1	Nichols et al. (1991), as presented in Cerco et al. (2010)
Biogeochemical parameters			
Selected water column rates^*^			
Phytoplankton growth rate constant		2.15 day^−1^	Feng et al. (2015)
POM solubilization rate constant		0.2 day^−1^	Feng et al. (2015)
Base-dissolved organic matter remineralization rate constant		0.00765 day^−1^	Feng et al. (2015)
Ratio of mol N:mol C for water column organic matter		0.15	Feng et al. (2015), Zimmerman and Canuel (2000)
Settling (sinking) velocity	Phytoplankton	0.1 m day^−1^	Feng et al. (2015)
	Small detritus	0.1 m day^−1^	Feng et al. (2015)
	Large detritus	5.0 m day^−1^	Feng et al. (2015)
	Aggregates	20 m day^−1^ (0.23 mm s^−1^)	Patten et al. (1966)
Critical bed shear stress of seabed organic matter		0.03 Pa	Assumed to be similar to seabed flocs; Cerco et al. (2010, 2013)
Erosion rate parameter for seabed organic matter		3 × 10^−5^ kg m^−2^ s^−1^	Assumed to be similar to seabed flocs; Cerco et al. (2010, 2013)
Partitioning of organic matter in river input		Varies in time based on output from the EPA watershed model	Irby and Friedrichs (2019)
Seabed rates			
Base remineralization rates of seabed organic matter		5.23 × 10^−4^ day^−1^	Zimmerman and Canuel (2000)
Coefficients for temperature–remineralization relationship	Base temperature	20 ^o^C	Testa et al. (2014)
	*Q*_10_	3	Testa et al. (2014)
Ratio of mol N:mol C in seabed organic matter		0.15	Zimmerman and Canuel (2001)
Seabed POM initialization		Spatially variable, based on observed seabed organic fraction	Zimmerman and Canuel (2001), Cerco et al. (2010)
Half saturation constant for O_2_ limitation in oxic respiration		6.25 μmol O_2_ L^−1^	Testa et al. (2014)
Half saturation constant for NO_3_ limitation in denitrification		1.0 μmol NO_3_ L^−1^	Laurent et al. (2016)
Half saturation constant for O_2_ limitation in nitrification		31.25 μmol O_2_ L^−1^	Testa et al. (2014)
Half saturation constant for O_2_ limitation in oxidation of ODUs		3.125 μmol O_2_ L^−1^	Testa et al. (2014)
Half saturation constant for O_2_ inhibition in denitrification		0.312 μmol O_2_ L^−1^	Testa et al. (2014)
Half saturation constant for O_2_ inhibition in anoxic mineralization		0.1 μmol O_2_ L^−1^	Laurent et al. (2016)
Half saturation constant for NO_3_ inhibition in anoxic mineralization		0.1 μmol NO_3_ L^−1^	Laurent et al. (2016)
Maximum nitrification rate		0.1 day^−1^	Testa et al. (2014)
Maximum oxidation rate of oxygen demand units		0.05 day^−1^	Testa et al. (2014)
Fraction of ODUs produced in the seabed that are solid and inert		0%	Laurent et al. (2016)
Base biodiffusion coefficients	Sediment and seabed organic matter	Surficial sediments—4.4 × 10^−11^ m^2^ s^−1^ Deep sediments—0 m^2^ s^−1^	Dellapenna et al. (1998)
	O_2_	11.05 × 10^−10^ m^2^ s^−1^	Laurent et al. (2016)
	NO_3_	9.78 × 10^−10^ m^2^ s^−1^	Laurent et al. (2016)
	NH_4_	9.803 × 10^−10^ m^2^ s^−1^	Laurent et al. (2016)
	ODU	9.7451 × 10^−10^ m^2^ s^−1^	Laurent et al. (2016)
Coefficients for temperature–biodiffusion relationship	Base temperature	20 °C	Testa et al. (2013)
	*Q*_10_ (particulates)	1.117	Testa et al. (2013)
	*Q*_10_ (solutes)	1.08	Testa et al. (2013)
Depths in the seabed where different biodiffusion coefficients are used for particulates	Surface coefficient	0–1 cm deep	Laurent et al. (2016)
	Deep coefficient	Over 3 cm deep	Laurent et al. (2016)
	Linear interpolation between surficial and deep values	1–3 cm deep	Laurent et al. (2016)

^*^Note that most water column biogeochemistry parameters are the same as Feng et al. (2015) and are not reprinted here, unless they are critical for the text

Modifying HydroBioSed so that inorganic sediment and resuspended organic matter affect light attenuation in the water column was critical for application to the Chesapeake Bay. This was neglected in earlier implementations (Moriarty et al. 2017, 2018), which focused on near-seabed processes. For this study, the concentrations of multiple classes of inorganic and organic sediment that are estimated by the sediment transport model are summed to estimate TSS, which is then used by the water column biogeochemical model in its estimate of light attenuation. Note that the same TSS value could be calculated from different concentrations of various particle classes. The diffuse light attenuation coefficient, *K*_D_, was estimated following Feng et al. (2015). Based on Cerco and Noel (2017), POM mass was assumed to equal 2.9 times the mass of POC.

### HydroBioSed Implementation for the Chesapeake Bay

Parameter values in the water column biogeochemistry, seabed biogeochemistry, and sediment transport routines were primarily based on Feng et al. (2015), Testa et al. (2014), and Cerco et al. (2010, 2013), respectively. Parameters that are new for this model implementation and/or are important for interpretation of our results are listed in Table 1 and briefly discussed here. Classes of inorganic sediment included sand, two classes of aggregated mud, and one class of unaggregated mud to represent the washload. Sediment parameter values are the same as in Cerco et al. (2010, 2013), except for the erosion rate parameter and critical shear stress for sand, which were adjusted using a series of sensitivity tests to match TSS observations from the Chesapeake Bay Program (CBP 2017). The need to adjust parameters is not unexpected because Cerco et al. (2010, 2013) and HydroBioSed use different formulations for sand erosion. Critical shear stresses for mud sediment classes were set to 0.03 Pa, which is low compared to values from other sites (e.g., Wu et al. 2018), but is consistent with studies from the Chesapeake Bay and its tributaries (Cerco et al. 2010, 2013; Dickhudt et al. 2009, 2011; Table 1). In addition to the plankton and detrital tracers previously included in the ECB model, HydroBioSed also accounts for an additional class of organic matter aggregates. Specifically, as phytoplankton and detritus are deposited on the seabed, they are incorporated into a seabed (particulate) organic matter class, which could later be entrained into the water column by resuspension. This seabed organic matter was assumed to have the same solubilization rate constant as large detritus when it was suspended, but was assigned a faster settling velocity (Table 1). Note that in ECB, POM is solubilized and then remineralized, but the remainder of this paper refers to the combination of these processes as “remineralization.”

Forcing for our coupled Chesapeake Bay model was based on previously published model implementations (Feng et al. 2015; Scully 2016; Irby and Friedrichs 2019). We used the curvilinear horizontal ChesROMS grid (Xu et al. 2012), which has an average horizontal resolution of 1.7 km inside the estuary with 20 vertical levels that are stretched to have increased resolution in surface waters and near the seabed. Temporally and spatially varying atmospheric forcing fields, including three-hourly winds with ~ 32 km resolution, were obtained from NCEP’s North American Regional Reanalysis (NARR) dataset. Open boundary conditions at the mouth of the estuary account for hourly changes in water level due to tides and subtidal variations using data from the Advanced Circulation Model (ADCIRC) EC2001 tidal database (Mukai et al. 2002) and observed water level from NOAA stations at Lewes, Delaware and Duck, North Carolina. Temperature and salinity concentrations at the open boundary were nudged to monthly climatological values from the 2001 World Ocean Atlas. Oxygen was nudged to be at 100% saturation at the open boundary, and radiation conditions were used for all other biogeochemical tracers.

Unlike previous versions of ChesROMS-ECB, this study also accounts for locally generated wind waves and open ocean swell because wave energy is important for suspended sediment within the Chesapeake Bay (e.g., Sanford 1994; Harris et al. 2012). Spatially and temporally varying estimates of wave height, period, direction, and orbital velocity were estimated using the Simulating WAves Nearshore model (SWAN; Booij et al. 1999). This study built on the SWAN implementation of Lin et al. (2002) for Chesapeake Bay by accounting for the propagation of ocean waves into the estuary. Specifically, the open boundary conditions at the bay mouth were set equal to three-hourly estimates from the National Oceanic and Atmospheric Administration’s Wave Watch III model (Tolman 2009). To account for the combined effect of waves and currents on modeled bed shear stresses, the hydrodynamic model (ROMS) used output from SWAN within ROMS’ implementation of Madsen's (1994) bottom boundary layer formulation as described by Warner et al. (2008). This parameterization accounts for wave-current interactions when computing the combined wave-plus-current-induced bed shear stresses.

Inputs of freshwater, sediment, and nitrogen from the watershed to the estuary were based on estimates from the Chesapeake Bay Program’s Watershed Model (phase 5.3.2; USEPA 2010; Shenk and Linker 2013), and carbon concentrations were derived from Tian et al. (2015), as in Irby and Friedrichs’s (2019) earlier ChesROMS-ECB implementation. These inputs included riverine sources of freshwater and both dissolved and particulate tracers, as well as inputs of freshwater and dissolved tracers from overland flow. Terrestrial inputs of POM were partitioned into phytoplankton, zooplankton, small detritus, and large detritus model variables based on Irby and Friedrichs (2019). Watershed inputs of sand and silt were input directly into the model as sand and large floc sediment classes, whereas clay was partitioned into washload (i.e., unaggregated mud) and small flocs, consistent with Cerco et al. (2013) (Table 1). Note that although the fine-grained sediment was classified as washload, small flocs, and large flocs, the model did not account for aggregation or disaggregation processes.

### Model Runs and Analysis

**Fig. 2:**
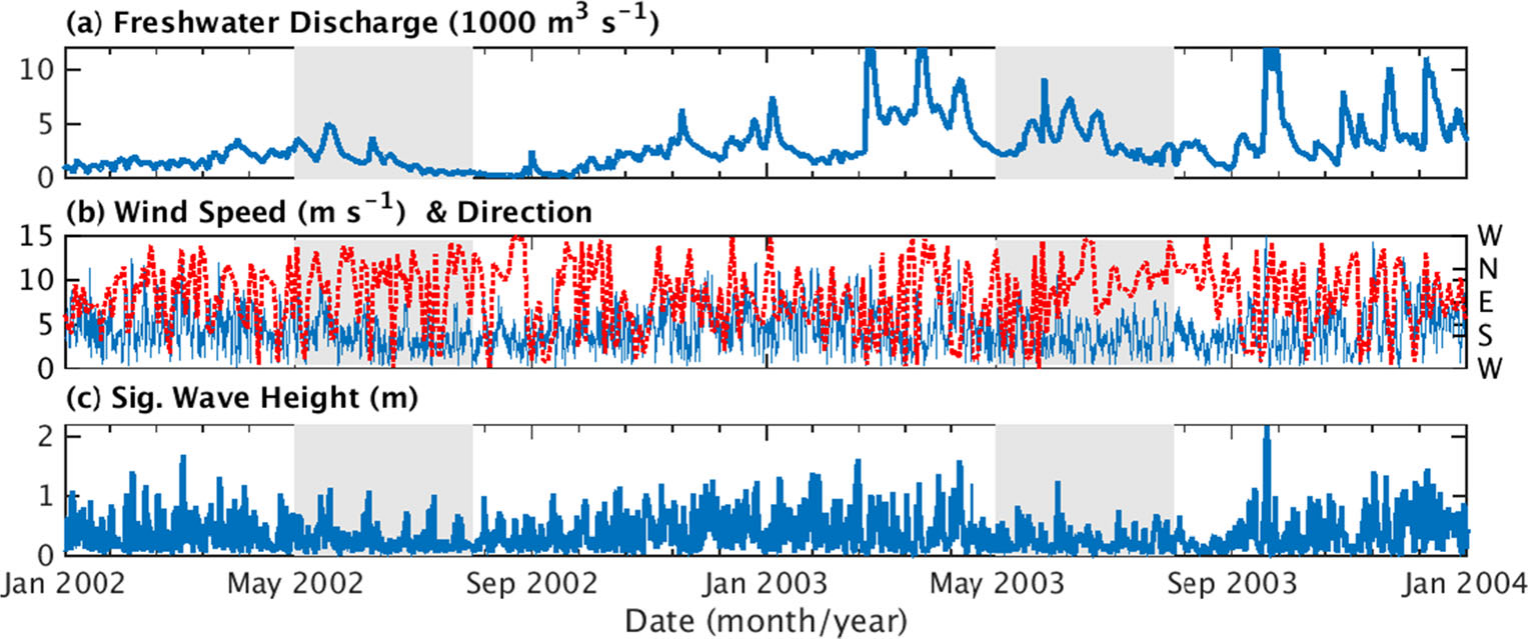
Time-series of model forcing, including **a** combined water discharge from tributaries and overland flow into the Chesapeake Bay from the EPA Watershed Model (USEPA 2010; Shenk and Linker 2013), (b) wind speed (blue line; left axis) and direction (red dots; right axis) toward which winds are blowing (in degrees clockwise from east) from NARR, and **c** significant wave height for a location outside the estuary at 20 m water depth estimated using SWAN (Booij et al. 1999). Shading indicates May–July 2002 and May–July 2003, the time periods of focus for this paper

A “Reference” model run using the HydroBioSed implementation described above was generated for the Chesapeake Bay to represent the years 2002, which was characterized by low-to-average riverine discharge, and 2003, which had high riverine inputs (Fig. 2; Cerco and Noel 2013). Initialization of hydrodynamic and water column biogeochemical fields for January 1, 2002, was taken from a multi-decadal model run from Irby and Friedrichs (2019). The initial seabed was based on spatially varying observations of grain size and organic fraction of particulates (Cerco et al. 2010; Zimmerman and Canuel 2001; Table 1; Fig. 1). Note that the muddy components of the seabed were assumed to contain 60% large flocs, 20% small flocs, and 20% unaggregated mud, consistent with Cerco et al. (2013). Following common practice (e.g., see Fennel et al. 2013; Feng et al. 2015), the coupled hydrodynamic–sediment transport–biogeochemical model was first run for the year of 2002, as a “spin-up” simulation. Output from January 1, 2003, from this “spin-up” model run was then used to initialize the Reference model run on January 2002. The model used a 15-s time step and daily averages were saved as output. Evaluation of our Reference model run focused on the subset of biogeochemical processes and concentrations most affected by resuspension, thereby complementing previous publications that evaluated hydrodynamics and biogeochemistry in the ChesROMS-ECB model (e.g., Feng et al. 2015; Irby et al. 2016).

Model analysis concentrated on the estuary’s thalweg, which includes the primary region where hypoxia occurs, and the early summer months of May–July. In particular, this study defined the “thalweg” as the along-estuary transect that transverses the deepest portion of the estuary (Fig. 1, Table 2). All “along-thalweg” distances are referenced to the northernmost end of the transect, i.e., “0 km along-thalweg” refers to the end of the transect offshore of Elkton, Maryland, in the Upper Bay (see Fig. 1). The thalweg was then broken into four regions based on each area’s qualitative response to resuspension with regard to primary productivity and remineralization: the Oligohaline, Upper, Mid, and Lower Bay regions (defined in Fig. 1). The months of May 1–July 31, 2002, and May 1–July 31, 2003, hereafter referred to as summer 2002 and summer 2003, were analyzed because oxygen concentrations are generally lowest in mid-summer (e.g., Bever et al. 2013), but conditions during the preceding months influence sediment and POC accumulation patterns in July (Fig. 2).

**Table 2:** Definitions of critical terms and acronyms

Term/acronym	Definition
Bottom water	Refers to model estimates 1 m above the seabed
Channel	Synonymous with thalweg. Note that other papers may use this term to refer to the relatively deep portion of the thalweg in the Upper and Mid Bay.
CSTMS	Community Sediment Transport Modeling System
ECB	Estuarine–carbon–biogeochemistry model
Formulation	Synonym for parameterization
HydroBioSed	The coupled hydrodynamic–sediment transport–biogeochemistry model used in this study
Main channel	See “Channel”
Model	A set of equations, e.g., those describing the physical and biogeochemical processes in HydroBioSed
Model run	An implementation of a model to represent a specific system (e.g., the Chesapeake Bay). Note that a user’s choice of parameters (e.g., remineralization rate constant, settling velocity) is chosen for individual model runs, but is not part of the model [equations].
No-Resuspension model run	This model run is identical to the Reference model run, except that resuspension was prevented from occurring by changing the erosion rate parameter, *M*, to zero.
Organic particulate	See POM
Parameter	A coefficient in an equation
Parameterization	An equation, or set of equations, that represents a specific process in a model
POC	Particulate organic carbon
POM	Particulate organic matter
Reference model run	Refers to the standard implementation of HydroBioSed used in this paper, as described in the “Methods” and Table 1
Remineralization	Refers to both solubilization and remineralization of POM for the purposes of this paper
ROMS	Regional Ocean Modeling System
RMSD	Root mean square difference, also referred to as the root mean square error (RMSE)
Sediment	Inorganic particles
Simulation	See model run
Surface water	Refers to model estimates 1 m below the atmosphere–ocean interface
SWAN	Simulating WAves Nearshore model
Thalweg	The line of deepest bathymetry along the length of the estuary. All “along-thalweg” distances are referenced to the northernmost end of the transect, i.e., “0 km along-thalweg” refers to the end of the transect offshore of Elkton, Maryland, in the Upper Bay (see Fig. 1).
TSS	Total suspended solids, i.e., the concentration of particulates, including sediment and POM, in the water column. It is calculated by summing the mass concentrations from each sediment and POM class in the model.

Model analysis focused on how seabed resuspension affected primary productivity and remineralization, as well as how changes in these processes affected oxygen and nitrogen dynamics. To isolate the role of resuspension on Chesapeake Bay biogeochemistry, results from the Reference model runs described above were compared to a second set of “No-Resuspension” simulations that were also run for 2002 and 2003. These No-Resuspension simulations were initialized based on output from January 1, 2002, and January 1, 2003 from the Reference model run, but resuspension was prevented by changing the erosion rate parameter (*M* in Eq. 1) to zero (see Warner et al. 2008). Differences between the No-Resuspension and the Reference model runs were used to evaluate how the entrainment of seabed material into the overlying water column affected primary productivity and remineralization, as well as oxygen and nutrient concentrations.

## Results

This section first compares the model estimates to observations from Chesapeake Bay and then characterizes the effect of resuspension on Chesapeake Bay biogeochemistry by comparing the Reference and No-Resuspension model runs. These first two sections of the “Results” primarily focus on summer 2003 for conciseness, but estimates from summer 2002 were similar, as shown in the final “Results” section. Note that “bottom water” and “surface water” refer to values 1 m above the seabed and below the atmosphere–ocean interface, respectively. Except where noted, means and standard errors were calculated using data from the 3-month time periods during summers 2002 and 2003, for analysis of both model estimates and observations. Variability in the results was estimated using 2 standard errors.

### Evaluation of the Reference Model Run

**Fig. 3:**
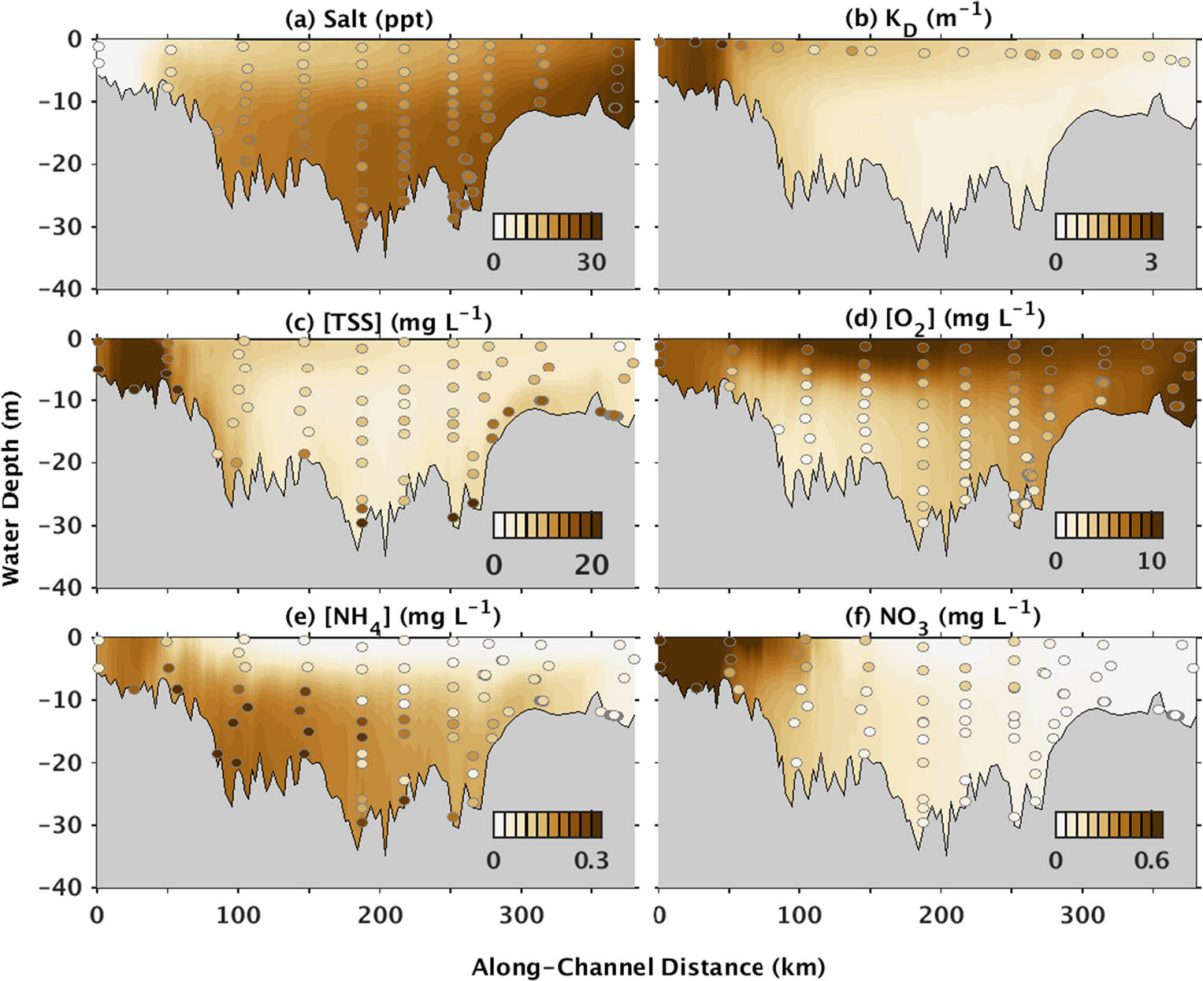
Transects of **a** salinity, **b** light attenuation coefficient *K*_D_, **c** TSS, **d** oxygen, **e** ammonium, and **f** nitrate. Estimates are from the Reference model run (shading) and Chesapeake Bay Program observations (CBP 2017; circles). Note that observed K_D_ values were averaged over the euphotic zone, i.e., from the water surface to the depth where light is 1% of surface values, by the Chesapeake Bay Program and so are located in the middle of the euphotic layer in the figure. Also, observed “nitrate” accounts for both nitrate and nitrite. Both observed and modeled data were averaged over May–July 2002 and 2003

Results from the Reference model run were compared with in situ observations of salinity, TSS, light attenuation, oxygen, ammonium, and combined nitrate+nitrite (hereafter referred to as nitrate) in summers 2002 and 2003 from the Chesapeake Bay Program (CBP 2017) (Fig. 3, Supplement A). Observations of primary production and oxygen consumption from 2002 to 2003 were unavailable, so these model estimates were compared to literature values.

The Reference model run generally reproduced the major observed along-thalweg patterns of salinity, TSS concentrations, and light attenuation coefficients (*K*_D_) from the Chesapeake Bay Program data (CBP 2017; Fig. 3a–c, Supplement A). In both the observations and modeled results, salinity ranged from near-zero in the Oligohaline region to ~ 30 in the Lower Bay. Additionally, in both datasets, TSS concentrations were highest in the ETM, compared to other regions of the estuary. The ETM in both datasets was located in the Oligohaline Bay at 39.2–39.4° N, i.e., ~ 10–50 km along the estuary’s thalweg in ~ 5–10 m water depth. Similar to TSS and salinity, both modeled and observed light attenuation coefficients (*K*_D_) were higher in the Oligohaline and Upper Bay compared to the Mid and Lower Bay (Fig. 3b). For summer 2003, in the Oligohaline Bay, i.e., where the ETM was located, *K*_D_ averaged 2.4 ± 0.1 m^−1^ in both the observations and model. In contrast, Mid Bay *K*_D_ was lower, averaging 1.0 ± 0.00 m^−1^ in the observations and 1.0 ± 0.1 m^−1^ in the model.

The Reference model run also captured the major along-thalweg gradients in oxygen, ammonium, and nitrate concentrations from the Chesapeake Bay Program data. Both the model and observations showed that oxygen and nitrate in the thalweg were relatively depleted, and that ammonium was relatively high below the pycnocline, compared to surface waters (Fig. 3d–f). Averaged bottom water oxygen concentrations in the thalweg, for example, ranged between 7.4 ± 0.05 mg L^−1^ in the observations and 4.3 ± 0.5 mg L^−1^ in the model, but were higher in surface waters for both the observations (8.6 ± 0.04 mg L^−1^) and the model (9.5 ± 0.2 mg L^−1^). Similarly, bottom water ammonium concentrations averaged 0.08 ± 0.002 mg L^−1^ in the observations and 0.19 ± 0.01 mg L^−1^ in the model. In surface waters, averaged ammonium concentrations decreased to 0.07 ± 0.002 mg L^−1^ in the observations and 0.06 ± 0.01 mg L^−1^ in the model.

Model estimates of primary productivity were compared to values derived from bottle incubations based on research cruises in summertime from 1982 to 2000 by Harding et al. (2002); observations from 2002 to 2003 were not available. Harding et al. (2002) estimated that maximum summertime production occurred between ~ 38.4 and 38.75° N, i.e., ~ 122–175 km along the estuary’s thalweg, with a mean of 0.39 ± 0.02 mg C L^−1^ day^−1^ during this 19-year period. The modeled maximum occurred in roughly the same location at 102 km along the estuary’s thalweg and had a similar magnitude (0.44 ± 0.08 mg C L^−1^ day^−1^; Table A7).

Finally, model estimates of oxygen consumption, which were calculated by summing aerobic remineralization and nitrification, were compared to estimates derived from incubation experiments at three locations along the estuary in the summers of 1989–1990 (Smith and Kemp 1995; Table A7). Measured values of oxygen consumption were not available for 2002–2003. Both the modeled and the observed summertime bottom water oxygen consumption peaked at 0.1–0.3 mg O_2_ L^−1^ h^−1^ at both ends of the thalweg, with lower values in the Mid Bay.

Overall, differences between model results and observations (Fig. 3, Supplement A) are inevitable considering the relatively coarse spatial and temporal resolution of the observational datasets, as well as the model’s necessary simplification or neglect of many processes such as aggregation (Tarpley et al. 2019) and seasonal succession of plankton communities (e.g., Malone and Ducklow 1990). The difference in age between decades-old observations (Harding et al. 2002; Smith and Kemp 1995) and modern model estimates from 2002 to 2003 also limited the evaluation of primary production and oxygen consumption. We note that additional observational studies focusing on processes affecting along-estuary and vertical variations in sediment transport and water column biogeochemistry would be particularly helpful for future improvements to the model’s skill.

Despite these limitations, the model reproduced the major observed spatial patterns in sediment and biogeochemical tracers, primary productivity, and oxygen consumption. Moreover, differences between the observations and standard model results imply that our estimates of the effect of resuspension on water column biogeochemistry are conservative. TSS concentrations in the model were generally lower than the observations, most notably in the near-seabed region of the Mid and Lower Bay regions (Fig. 3). Additionally, oxygen and ammonium concentrations were biased high and low, respectively, with the greatest model–observation differences in the Mid Bay (Fig. 3). As further discussed in later sections of the paper, these results together imply that the model generally underestimated near-bed remineralization rates, oxygen consumption, and ammonium production. This ultimately resulted in increased oxygen concentrations and decreased ammonium concentrations compared to the observations. Overall, this implies that our estimates of resuspension-induced changes in these regions are conservative, reinforcing our conclusion that resuspension can impact the Bay’s biogeochemical dynamics.

### Effect of Resuspension on Sediment, POC, O_2_, and NH_4_

**Fig. 4:**
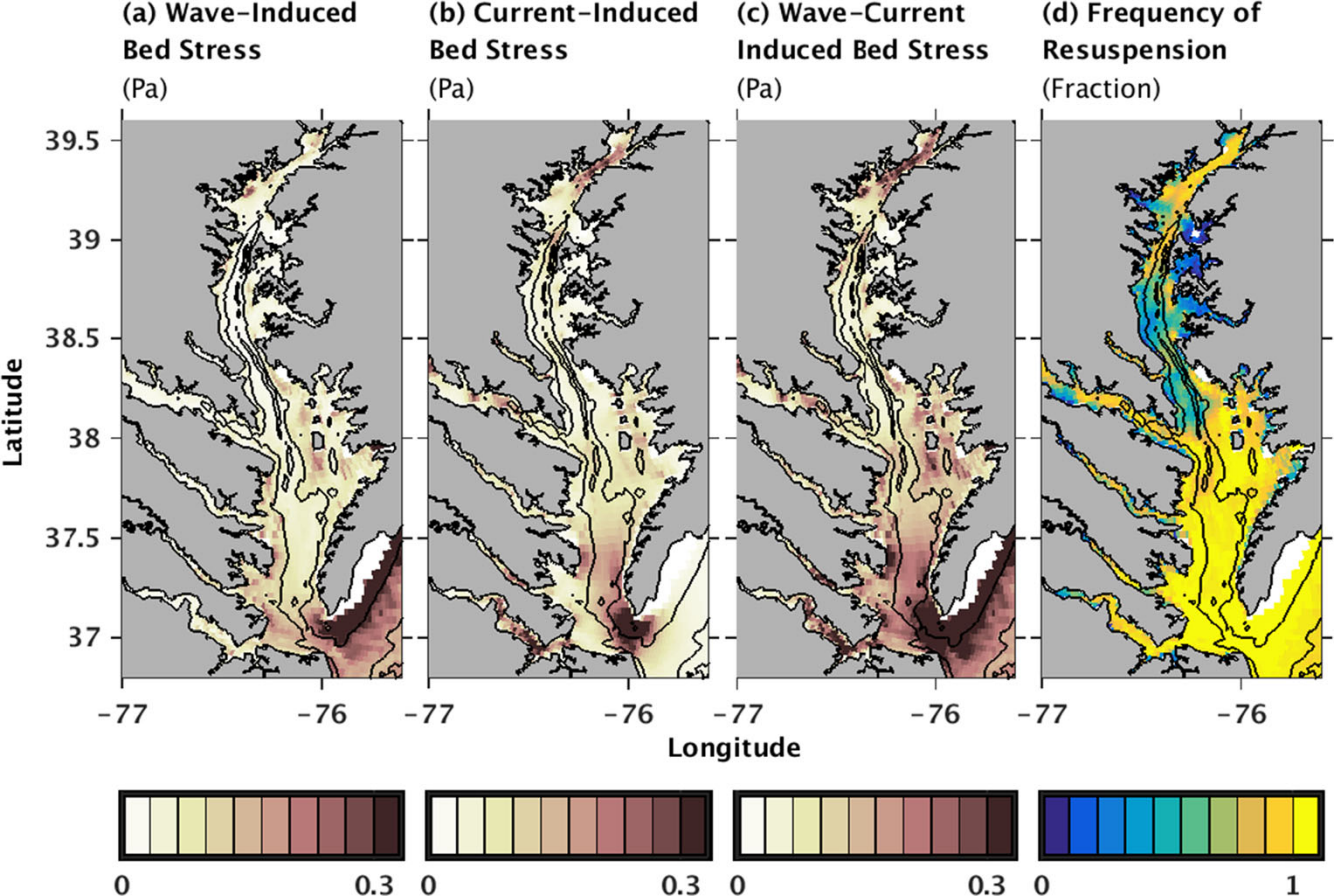
Estimates from the Reference model run of the magnitudes of **a** wave-induced bed shear stress, **b** current-induced bed shear stress, and **c** combined wave-plus-current-induced bed shear stresses, all averaged over May–July 2003. **d** Fraction of time in July 2003 when the combined wave-plus-current-induced bed shear stresses exceeded 0.03 Pa, the critical threshold for resuspension of mud and particulate organic matter

Estimates from the Reference model run for summer 2003 indicated that bed stresses, which along with sediment availability determines where particulates may be resuspended, were highest in the Oligohaline Bay and Lower Bay, with a minimum in the Upper Bay (Fig. 4a–c). In the Oligohaline Bay throughout most of summer 2003, fast tidal currents and riverine-influenced flows created bed stresses that consistently exceeded 0.03 Pa, the assumed threshold for erosion of mud and organic matter (Table 1; Fig. 4b, d). Near-bed current speeds decreased in the Upper Bay, however, reducing bed stresses such that this threshold was only exceeded about half of the time. In the Lower Bay and southern portion of the Mid Bay, tidal and wave energy were higher, producing modeled bed shear stresses that nearly always exceeded 0.03 there (Fig. 4c, d).

**Fig. 5:**
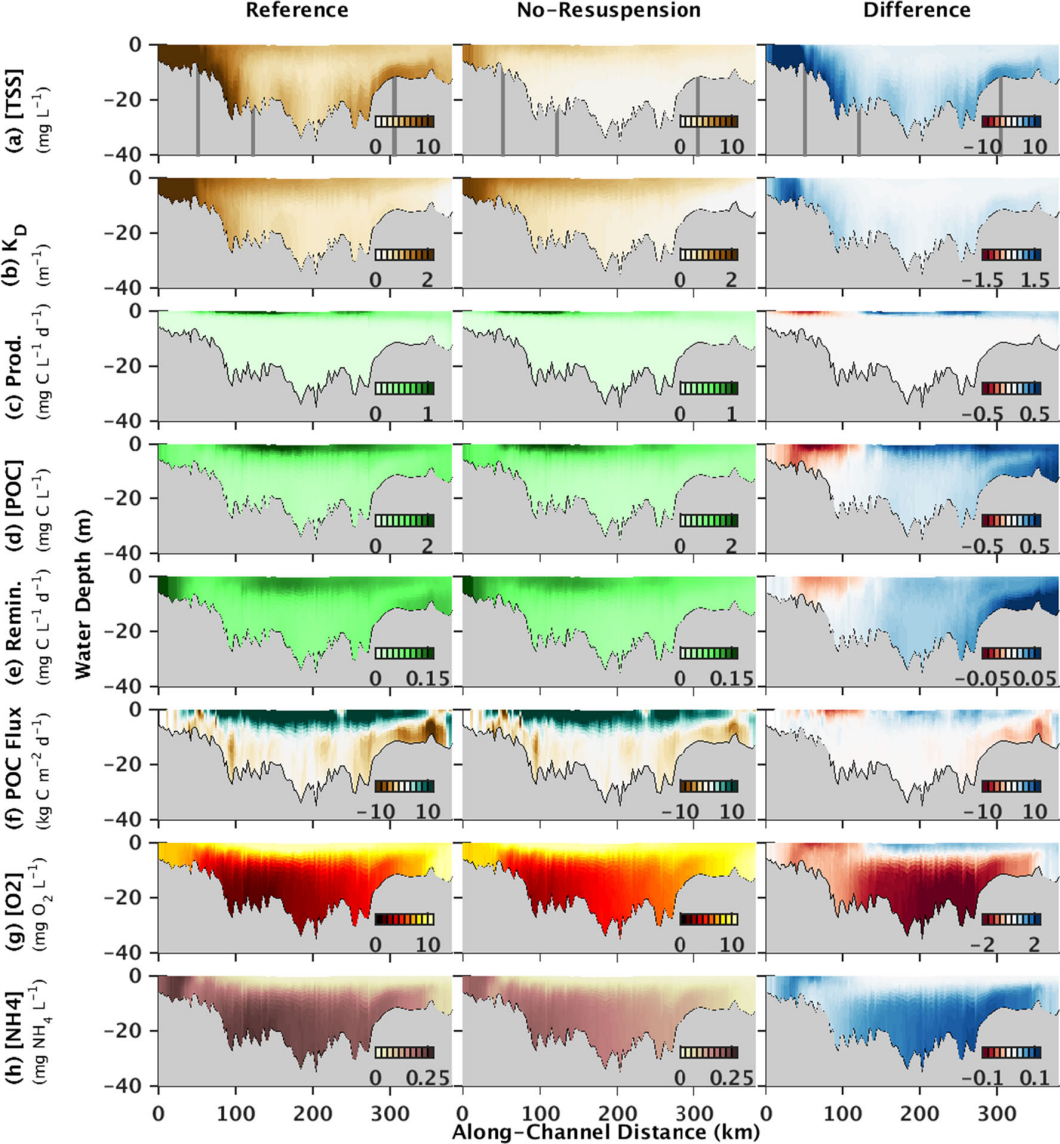
Estimates from the Reference (left) and No-Resuspension (center) model runs for a transect along the thalweg of the Chesapeake Bay (location given in Fig. 1a). The change induced by resuspension (right) is calculated by subtracting estimates from the No-Resuspension model run from those from the Reference model run. All estimates were averaged over May–July 2003. Panels include **a** TSS concentration, **b** light attenuation coefficient (*K*_D_), **c** primary productivity (Prod), **d** POC concentration, **e** POC remineralization (Remin), **f** along-estuary POC fluxes, and concentrations of **g** oxygen and **h** ammonium. In the top panels, the gray vertical lines indicate the boundaries between the Oligohaline, Upper, Mid, and Lower Bay

Comparing results from the summer 2003 Reference and No-Resuspension model runs revealed that resuspension induced by these energetic bed stresses increased TSS concentrations throughout the Chesapeake Bay. In the Oligohaline Bay’s surface waters, for example, surface TSS concentrations reached up to ~ 20 mg L^−1^ in the Reference model run, but only ~ 7 mg L^−1^ in the No-Resuspension model run (Fig. 5a), and the differences were greater at depth. On average, surface water in the Oligohaline Bay increased by 190 ± 4.5%. In the Upper and Mid Bay, resuspension enhanced TSS to a lesser extent compared to the Oligohaline Bay, but near-bed concentrations in these more southern regions still increased by an average of about 5 ± 0.03 mg L^−1^, or 663 ± 2.2% (Fig. 5a). Overall, resuspension-enhanced turbidity increased light attenuation throughout the water column. In surface waters of the Oligohaline Bay during summer 2003, the diffuse light attenuation coefficient, *K*_D_, reached up to 2.7 m^−1^ in the Reference model run, compared to 1.8 m^−1^ in the No-Resuspension model run, with an average increase of 23 ± 0.45% (Fig. 5b).

Accounting for resuspension in the model reduced primary productivity in the Oligohaline and Upper Bay where TSS concentrations and light attenuation increased the most, but enhanced production in surface waters of the Mid and Lower Bay in summer 2003 (Fig. 5c). In the Oligohaline and Upper Bay regions, i.e., north of 120 km along the estuary’s thalweg, resuspension reduced phytoplankton production in the surface waters of the thalweg in summer 2003 by a mean of − 0.18 ± 0.04 mg C L^−1^ day^−1^, or − 26 ± 0.35% (Fig. 5c). In the surface waters of the Lower Bay thalweg, in contrast, primary production increased by ~ 0.32 ± 0.02 mg C L^−1^ day^−1^, or 76 ± 0.54% (Fig. 5c).

The effect of resuspension on POC concentrations, remineralization, and along-estuary advective POC fluxes also varied along the length of the estuary in summer 2003 in the model (Fig. 5d–f). In the Oligohaline and Upper Bay, resuspension decreased bottom water POC concentrations by an average of − 0.1 ± 0.01 mg C L^−1^, or − 12 ± 0.35%, in the thalweg (Fig. 5d). In contrast, resuspension increased bottom water POC concentrations in the Lower Bay thalweg from ~ 0.5 ± 0.02 mg C L^−1^ in the No-Resuspension model run to ~ 0.8 ± 0.05 mg C L^−1^ in the Reference model run, with an average change of 87 ± 0.40% (Fig. 5d). Similar to resuspension-induced changes in POC concentrations, resuspension caused the magnitude of along-estuary POC fluxes, as well as remineralization, to decrease in the Upper Bay thalweg (Fig. 5e, f). In this region, both down-estuary POC fluxes in the surface waters and up-estuary POC fluxes in bottom waters decreased in the Reference model run compared to the No-Resuspension model run (Fig. 5f). Bottom water remineralization decreased by up to ~ − 0.01 mg C L^−1^ day^−1^, or 13%, when the model run accounted for resuspension (Fig. 5e). In the Lower Bay thalweg, in contrast, resuspension increased the magnitude of along-estuary POC fluxes in both surface and bottom waters, as well as remineralization (Fig. 5e, f). Overall, these resuspension-induced changes in POC concentrations, fluxes, and remineralization were generally co-located with shifts in primary production (Fig. 5c–f) in that all of these variables decreased in the Oligohaline and/or Upper Bay and increased in the Lower Bay. However, resuspension-induced changes in POC concentrations, fluxes, and remineralization occurred throughout the water column, whereas large changes in primary production were limited to surface waters in the model. Additionally, the largest increase in POC remineralization was co-located with the resuspension-induced increase in upstream POC fluxes in the bottom water column of the Mid to Lower Bay (Fig. 5c–f).

Including resuspension in the model decreased oxygen concentrations throughout almost the entire thalweg in summer 2003 (Fig. 5g). The largest reduction in oxygen levels occurred below the pycnocline in the Mid Bay, where bottom water concentrations decreased by up to 2.2 mg O_2_ L^−1^, with a mean decrease of − 1.9 ± 0.06 mg O_2_ L^−1^, or − 45 ± 0.16%, in the Reference model run compared to the No-Resuspension model run (Fig. 5g). In the Oligohaline and Upper Bay, in contrast, the largest reduction in oxygen levels occurred in surface waters, where concentrations decreased by a maximum of 1.5 mg O_2_ L^−1^, with an average change of − 7.8 ± 0.08%. Resuspension also decreased oxygen concentrations to a lesser extent in the bottom waters of the Oligohaline, Upper, and Lower Bay (Fig. 5g). Resuspension only increased oxygen levels in the surface waters of the Mid and Lower Bay, where concentrations increased by up to 1.1 mg O_2_ L^−1^, with an average increase of 6.7 ± 0.05%, when averaged over summer 2003 (Fig. 5g).

In contrast to oxygen, accounting for resuspension in the model caused ammonium concentrations to increase throughout the thalweg in summer 2003 (Fig. 5h). The largest increases were estimated to occur below the pycnocline in the Mid and Lower Bay, where bottom water ammonium concentrations increased by up to ~ 0.09 mg N L^−1^, with an average increase of 74 ± 0.60%, in the Reference model run compared to the No-Resuspension model run (Fig. 5h). Ammonium levels also increased in the Oligohaline Bay where concentrations in surface waters similarly increased by up to ~ 0.07 mg N L^−1^, with an average increase of 93 ± 1.3% (Fig. 5h). In contrast, resuspension caused no change in surface water ammonium levels in the Mid and Lower Bay, where concentrations were near-zero.

### Interannual Variability of the Effect of Resuspension on Water Column Biogeochemistry

**Fig. 6:**
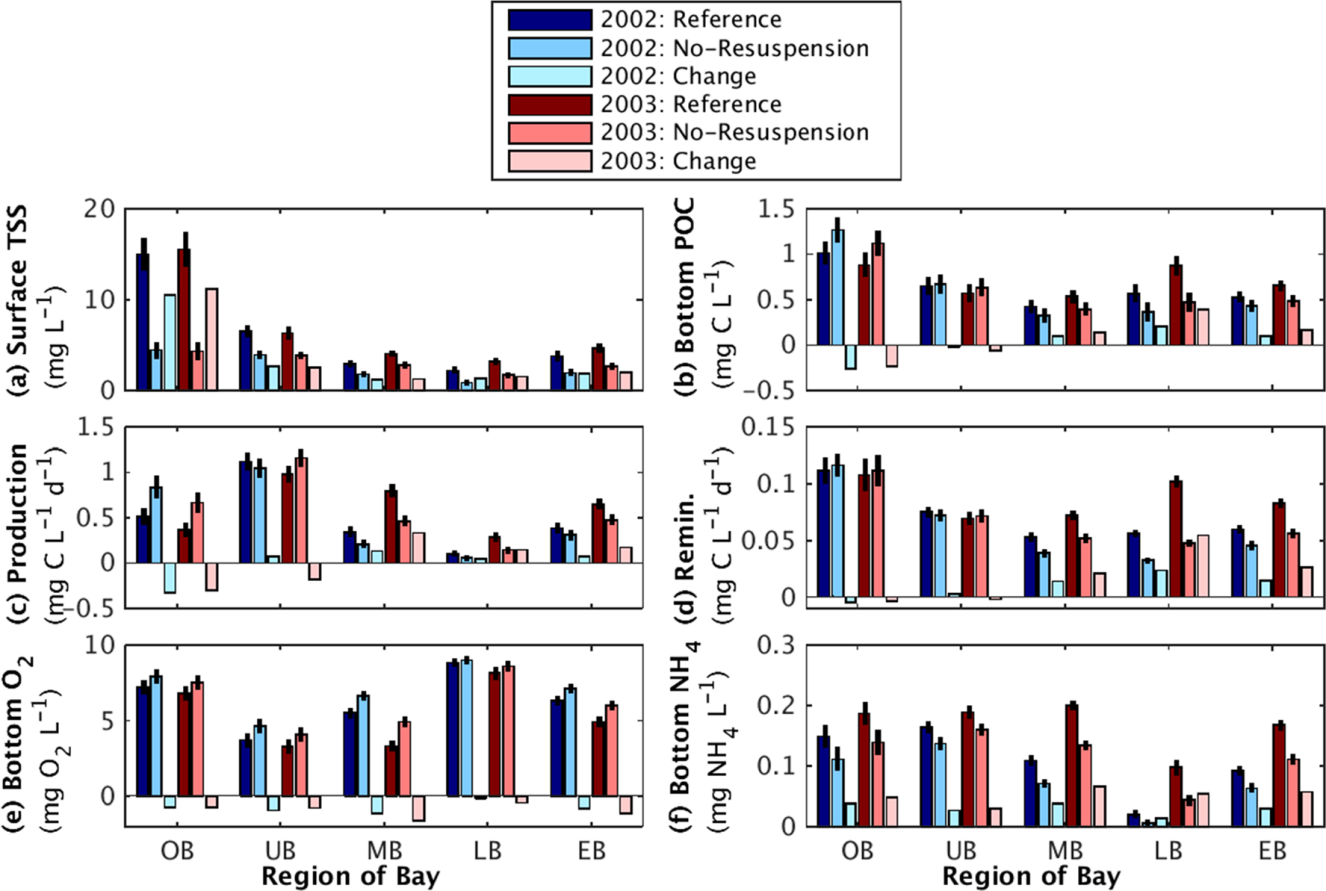
Bar charts of biogeochemical rates and concentrations estimated by the Reference (dark blue and dark red bars) and No-Resuspension (medium blue and medium red bars) model runs. All estimates were temporally averaged over July 2002 or July 2003 and spatially averaged for the grid cells within different regions of the thalweg (Fig. 1c). Bars represent the Oligohaline Bay (OB), Upper Bay (UB), Mid Bay (MB), Lower Bay (LB), and the entire Chesapeake Bay thalweg (EB). For each region, the difference between the averaged values for the Reference and No-Resuspension model runs was indicated by the light blue and light red bars. Estimates are for **a** surface water TSS concentration, **b** bottom water POC concentration, **c** surface water primary productivity, **d** bottom water remineralization, **e** bottom water O_2_ concentration, and **f** bottom water NH_4_ concentration. Black vertical lines indicate two standard errors of estimates over each specific time period and region

The effect of resuspension on TSS, POC concentrations, primary production, remineralization, oxygen, and ammonium was similar in the summers of 2002 and 2003 (Fig. 6; Supplement B). Note that this is true even though summer 2002 and summer 2003 followed relatively dry (i.e., low-discharge) and wet (i.e., high-discharge) springs, respectively (Fig. 2). In all regions of the thalweg except the Upper Bay, the resuspension-induced changes in the model estimates were in the same direction (positive or negative) during both 2002 and 2003. In contrast, the model results indicated more interannual variability in the Upper Bay, where resuspension-induced effects on primary productivity, POC concentrations, and remineralization transitioned from decreases in the Oligohaline Bay to increases in the Mid Bay (Fig. 6). Additionally, in almost all regions and time periods considered, the difference between the means of the Reference and No-Resuspension model runs exceeded the variability within each simulation, as defined by two standard errors. This change due to resuspension also generally exceeded the interannual variations. Specifically, the resuspension-induced changes were larger than the difference between model estimates for 2002 versus 2003 for 66% of the regions and variables considered here. In 20% and 8% of the cases, the change due to resuspension exceeded the interannual variability by a factor of at least 2 and 5, respectively. Overall, this analysis indicated that resuspension induced significant changes in Chesapeake Bay biogeochemistry that were consistent in both high-discharge and low-discharge years (Fig. 6).

Although the spatial shifts in water column biogeochemistry due to resuspension were similar in 2002 and 2003, the magnitude of the changes was generally larger during the high-discharge year, compared to the low-discharge year (Fig. 6). Overall, almost two-thirds of the regions and variables considered in Fig. 6 showed larger percent changes due to resuspension in 2003 compared to 2002. Primary productivity experienced the most interannual variability in its response to resuspension; resuspension-induced changes in surface water primary productivity varied by ~ 12% between years, averaged over the different regions. In contrast, spatially averaged interannual variability of resuspension-induced changes for other variables ranged from 5 to 8%. No one region had persistently more interannual variability than the others.

## Discussion

**Fig. 7:**
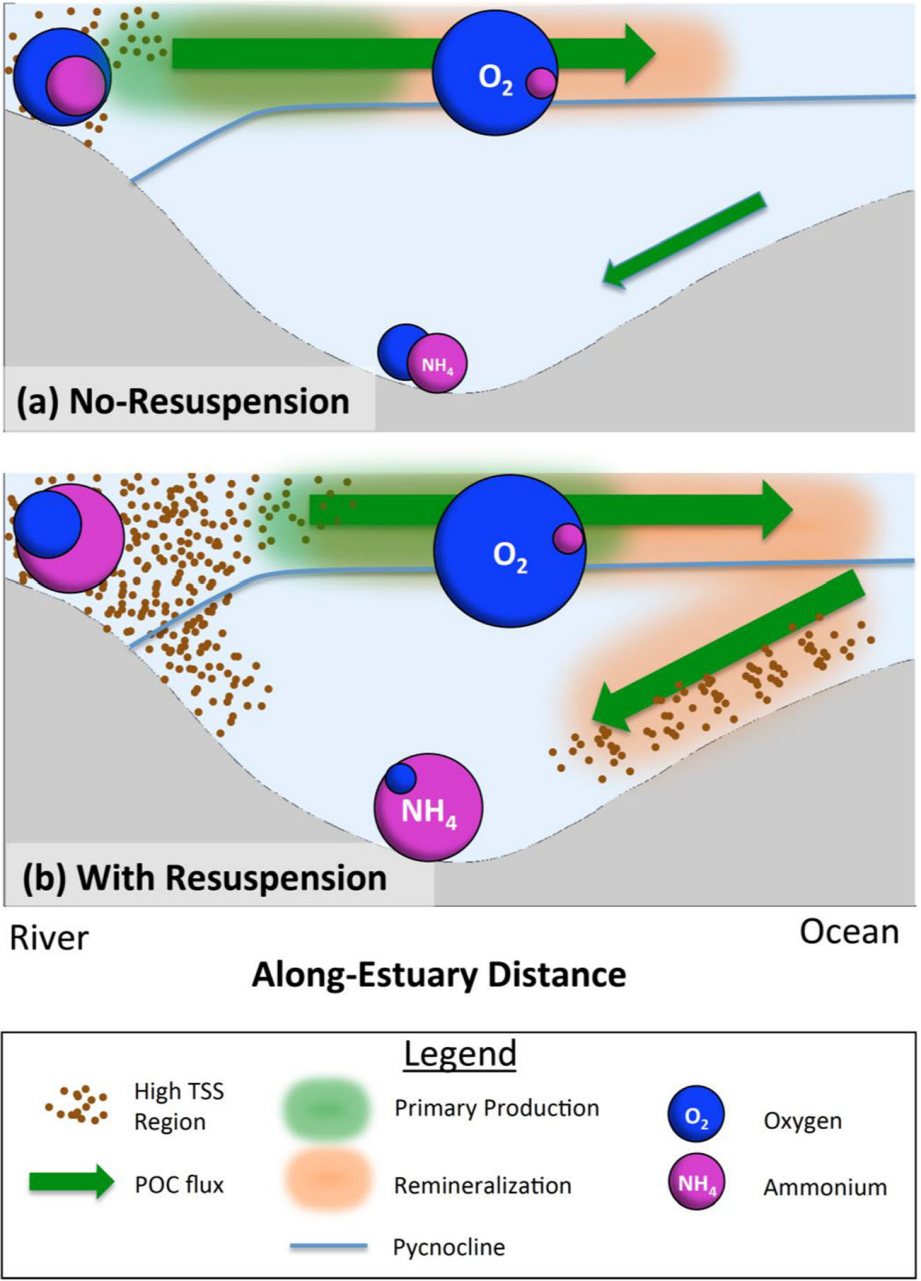
**a, b** Schematic of how resuspension affects biogeochemical processes based on HydroBioSed model estimates for Chesapeake Bay

Overall, the model experiments described above quantify the extent to which resuspension can cause a down-estuary shift in primary production, POC concentrations, and remineralization, causing a decrease in bottom water oxygen concentrations and an increase in ammonium concentrations throughout almost the entire Chesapeake Bay channel (Fig. 7). These along-estuary variations occur during both high-discharge (2003) and low-discharge (2002) years. This section synthesizes and explores the variability in resuspension-induced changes in primary production and remineralization, as well as oxygen and ammonium concentrations, before considering implications for future studies.

### Along-Estuary Variability in the Response of Primary Productivity and Remineralization to Resuspension

The response of primary production to resuspension varies along the Chesapeake Bay thalweg, depending on whether phytoplankton growth is primarily light-limited or nutrient-limited. In the Oligohaline Bay, where phytoplankton growth is primarily light-limited (e.g., Harding 1994; Harding et al. 2002), resuspension-induced increases in turbidity are sufficient to reduce organic matter production (Figs. 5c and 7). These results are consistent with observations indicating that TSS is the main factor in determining light attenuation in the northernmost portion of the Bay (Xu et al. 2005). Note that the resuspension-induced decrease of phytoplankton growth in the Oligohaline Bay reduces nutrient uptake there, so that more dissolved inorganic nitrogen flows downstream to the surface waters of the Upper to Mid Bay. Unlike in the Oligohaline Bay, phytoplankton growth in the Mid Bay is primarily nutrient-limited (Harding 1994; Harding et al. 2002). Therefore, the resuspension-induced increase in the delivery of nutrients from the Oligohaline Bay stimulates primary productivity in the Mid Bay (Fig. 5c). In summary, the resuspension-induced increase in turbidity shifts the locus of primary production downstream, from the Upper and Mid Bay to the Mid Bay. These model results build on studies from other estuaries showing that primary production peaks downstream of turbid regions (Cloern 1987; e.g., Delaware: Pennock and Sharp 1986) by demonstrating the importance of resuspension in addition to riverplume delivery for light attenuation in estuaries.

The location of the transition from regions where resuspension decreases primary production, i.e., the Oligohaline Bay, to regions where resuspension increases primary production, i.e., the Mid Bay, will shift depending on environmental conditions. In summer 2003, this transition occurred at ~ 38.75° N, i.e., ~ 120 km along the thalweg, as surface water TSS and ammonium concentrations decreased in the down-estuary direction (Fig. 5), and phytoplankton growth changed from primarily being light-limited to nutrient-limited. In contrast, the location of this transition was farther upstream in summer 2002. This interannual variation in the impact of resuspension on primary production was likely due to a reduction in river discharge and the extent of the turbid freshwater plume in 2002 compared to 2003 (Figs. 2 and 6). This result is consistent with previous studies in the Chesapeake Bay and other systems showing the importance of river discharge on primary production (e.g., Pennock and Sharp 1986; Harding 1994; Harding et al. 2005; Roman et al. 2005; McSweeney et al. 2016; Da et al. 2018), but our results additionally emphasize that river discharge affects the transport of resuspended sediment, thereby influencing light attenuation and primary production. Furthermore, our result that TSS concentrations and light attenuation were sensitive to resuspension (Fig. 5a, b) implies that variations in resuspension magnitude or frequency, due to variability in river discharge or other factors, can also affect the extent to which phytoplankton growth is primarily light-limited and impact the location of the transition from light-limited to nutrient-limited production. TSS concentrations in the Oligohaline Bay change in response to resuspension, sediment properties, and seasonally varying wave energy (Sanford 1994; Sanford et al. 2001; Harris et al. 2012; Fig. 5a). In the surface waters of the Oligohaline Bay, for example, an increase of TSS from 15 to 30 mg L^−1^ causes the model’s *K*_D_ to increase from 1.78 to 2.72 m^-1^ (see equation 3 in Feng et al. 2015; assuming a salinity of 10), and primary production to decrease by 30%, from 0.016 to 0.021 mg C L^−1^ day^−1^ (see eqs. in Tables A1, A3, and A4 in Feng et al. 2015; assuming 80 W m^−2^ of incident light).

Concentrations of POC and remineralization respond to resuspension indirectly, i.e., due to changes in light attenuation and primary productivity, as well as directly, i.e., as seabed organic matter is entrained into the water column. In the Oligohaline Bay, the resuspension-induced decrease in organic matter production, due to light attenuation as described above, is only partially offset by the increase in POC concentrations due to entrainment of seabed material into the water column. Thus, POC concentrations, as well as remineralization, decrease in this northernmost portion of the Bay (Fig. 5d, e). In the Mid to Lower Bay channel, in contrast, resuspension increases POC concentrations by (1) enhancing primary production because of higher nutrient supply from upstream, (2) entraining local material from the seabed into the water column, and (3) facilitating import of POC from the Lower Bay to the Mid Bay (Figs. 4 and 5c, f and 7). This resuspension-induced enhancement of POC concentrations in the Mid to Lower Bay channel increases remineralization in this region. Overall, the result that POC production, resuspension, and transport patterns affect remineralization rates is consistent with studies from other regions (e.g., Lampitt et al. 1995; Capet et al. 2016; Moriarty et al. 2018), but expands on previous results by considering the spatially variable roles of resuspension-induced changes in primary production versus remineralization.

### Implications for Oxygen and Nitrogen Dynamics

Resuspension-induced changes in bottom water oxygen and ammonium concentrations in the model are driven by primary production in the Oligohaline Bay and by remineralization in the Mid Bay. This result is driven by POC transport patterns, which cause a spatial offset between POC production versus accumulation and remineralization (Fig. 7). This offset causes changes in water column biogeochemistry to be dominated by photosynthesis in the Oligohaline Bay, where not all POM that was produced accumulates. In contrast, changes in water column biogeochemistry in the bottom waters of the Mid and Lower Bay, where POC is imported from the surface layer and/or the southern portion of Chesapeake Bay, are dominated by remineralization.

As a result of the varying influence of photosynthesis and remineralization along the estuary, resuspension decreases bottom water oxygen concentrations and increases bottom water ammonium concentrations throughout the entire thalweg in the model (Fig. 7). Specifically, in the Oligohaline Bay, resuspension reduces oxygen concentrations primarily via the turbidity-induced decrease in photosynthesis. Resuspension has a lesser impact on oxygen consumption via remineralization in the Oligohaline Bay because a portion of the POM produced in this region is exported. In the Mid Bay, in contrast, bottom water oxygen concentrations are more sensitive to remineralization of available POM than to photosynthesis or primary production. Thus, the resuspension-induced increase in POM and remineralization increases oxygen consumption in the Mid Bay, lowering bottom water oxygen concentrations there. Consistent with patterns of oxygen dynamics, ammonium concentrations in the Oligohaline Bay increase in response to reduced phytoplankton growth, which lowers nutrient uptake rates (Figs. 5, 6, and 7). In the Mid Bay, remineralization of resuspended organic matter produces ammonium, increasing concentrations in bottom waters in this region.

The response of oxygen and ammonium concentrations to changes in primary production and remineralization described above is consistent with the literature when results are averaged over the entire thalweg, but our model results build on previous studies by demonstrating the inherent spatial variability caused by estuarine circulation. Generally, reductions in phytoplankton productivity and organic matter concentrations are expected to increase oxygen levels due to decreased remineralization rates (e.g., Bricker et al. 2007; Kemp et al. 2009; and references therein). This expectation has motivated management programs across the globe to reduce nutrient inputs to coastal watersheds (e.g., Rabalais et al. 2010). Consistent with these expectations, our model results demonstrate that, when averaged over the entire thalweg, resuspension-induced increases in phytoplankton productivity, POC concentrations, and remineralization cause lower oxygen concentrations. Yet, in some localized regions, i.e., in the Oligohaline Bay where more POC is produced than is remineralized, our model results showed that resuspension-induced decreases in primary production are co-located with decreases in oxygen concentrations. This implies that systems such as the Chesapeake Bay may experience spatially variable responses to management actions that affect turbidity and sediment transport patterns. For example, reducing riverine sediment inputs may increase photosynthesis, primary production, and oxygen concentrations in the Oligohaline Bay. This increased primary production in the northernmost portion of the Bay, however, could result in increased export of POC from this region to the Mid Bay, thereby enhancing remineralization and oxygen consumption downstream. Accounting for such spatial variability is critical for understanding how individual parts of systems like the Chesapeake Bay may respond to management decisions, and our results emphasize that a coastal system’s response to management efforts may vary locally.

### Implications for Future Studies and Environmental Management

Accounting for resuspension improves the model’s representation of observed patterns of turbidity and primary production. When HydroBioSed neglects resuspension, riverine sediments are quickly deposited and no ETM forms, causing primary production to peak closer to the Susquehanna River mouth (Figs. 5 and 7). Including resuspension in the model allows an ETM to form and decreases light levels, especially in the Oligohaline Bay. This causes primary productivity to shift downstream, so that the model better represents observations (Sanford et al. 2001; Harding et al. 2002). This implies that accounting for resuspension in observational and modeling studies is likely also important for understanding biogeochemical dynamics in other estuaries and coastal regions where turbidity affects primary production (Cloern 1987; e.g., Delaware Bay: Pennock and Sharp 1986; McSweeney et al. 2016). Moreover, a better understanding of processes such as seabed consolidation and particle aggregation that affect spatial variability in resuspension and TSS would be useful for further improving model skill (Tarpley et al. 2019; Moriarty et al. 2014).

Differences between the observations and standard model results imply that our estimates of the effect of resuspension on water column biogeochemistry are conservative. TSS concentrations in the model are generally lower than the observations, most notably in the near-seabed region of the Mid and Lower Bay regions (Fig. 3). Additionally, oxygen and ammonium concentrations are biased high and low, respectively, with the greatest model–observation differences in the Mid Bay (Fig. 3). Together, these results imply that the model’s underestimation of resuspension in the Lower and Mid Bay regions, and the associated underestimation of the up-estuary flux of POC, caused an underestimation of remineralization rates, oxygen consumption, and ammonium production in these regions. This ultimately resulted in increased oxygen concentrations and decreased ammonium concentrations compared to the observations. Overall, this implies that our estimates of resuspension-induced changes in these regions are conservative, reinforcing our conclusion that influxes of POC into this region enhance remineralization rates there, lowering oxygen and increasing ammonium concentrations.

A few Chesapeake Bay biogeochemistry models account for some processes relating to resuspension and subsequent redistribution of POM, but our results can help refine their parameterizations. For example, a previous model by Cerco et al. (2013) facilitates the accumulation of POM in the channel as opposed to the shoals by using water-depth-dependent values for POM settling velocities so that particulates settle more slowly in shallow areas compared to deeper areas. An alternative parameterization by Feng et al. (2015) prevents POM deposition and burial when bed stresses are high. Based on our model results, these two alternate parameterizations may underestimate transport of POM, however, because neither allows organic material to be resuspended once it is deposited. Additionally, results from our model indicate that accounting for the influence of waves on bed shear stress is important for estimating resuspension in the Mid and Lower Bay (Fig. 4b, c), implying that parameterizations such as Feng et al. (2015), which only accounts for current-induced bed stresses, may be further underestimating transport of POM. Without running a full sediment transport–biogeochemistry model as we did, future parameterizations could consider adjusting POM settling velocities based on bed stress patterns, as opposed to water depth; account for wave-induced bed stresses; and allow seabed organic matter to be resuspended, e.g., similar to the parameterization of Feng et al. (2015) for inorganic sediment. Note, however, that these recommendations would require testing before implementation in other Chesapeake Bay models. Additionally, these revised parameterizations would still neglect variations in seabed erodibility and therefore resuspension, so the use of HydroBioSed or other models that account for temporary storage of POM in the seabed (e.g., Capet et al. 2016) is recommended when resuspension and redistribution of particulates has a large effect on biogeochemistry.

Observational studies from different locations (e.g., Lampitt et al. 1995; Abril et al. 1999; Queste et al. 2016; Zeng et al. 2017; Niemistö et al. 2018; Porter et al. 2018; Bianucci et al. 2018), as well as recent modeling efforts for different environments (e.g., this study; Moriarty et al. 2017, 2018; Lajaunie-Salla et al. 2017), have indicated that the impact of resuspension on biogeochemical processes varies among systems. In Chesapeake Bay, for example, our results showed that vertically varying, bidirectional fluxes of POC due to estuarine circulation impacted the effect of resuspension on Bay biogeochemistry (see “Along-Estuary Variability in the Response of Primary Productivity and Remineralization to Resuspension” and “Implications for Oxygen and Nitrogen Dynamics” sections). Other processes may be significant in systems whose characteristics differ from Chesapeake Bay in terms of river discharge or bathymetry. Consideration of additional sites, as well as time periods characterized by different environmental conditions such as storms, will increase understanding of how resuspension affects water column biogeochemistry. This will also lead toward a better understanding of when interactions among hydrodynamic, sediment transport, and biogeochemical processes are important to consider in observational studies, and when a coupled hydrodynamic–sediment transport–biogeochemistry model is needed in lieu of a more simplified model.

In conclusion, accounting for sediment processes, including resuspension, should be considered when evaluating the effects of management actions on water quality in the Chesapeake Bay, as well as other estuarine and coastal systems. Notably, resuspension decreased oxygen and increased ammonium bottom water concentrations throughout the Bay’s channel. This result occurred due to the transport of organic matter from the northern to the southern Bay via estuarine circulation, as well as decreased primary productivity in the northern Bay and increased organic matter remineralization in the central Bay. Averaged over the Chesapeake Bay thalweg in summer 2003, resuspension decreased concentrations of oxygen by ~ 25% and increased ammonium by ~ 50% in the bottom water column. Overall, changes in water column biogeochemistry due to resuspension were of the same order of magnitude and generally exceeded both short-term variability in model results during individual summers and interannual variability between the years 2002 (a dry year) and 2003 (a wet year).

## Electronic Supplementary Material

ESM 1 (DOCX 67 kb)

ESM 2 (DOCX 400 kb)

## Data Availability

Model datasets are publicly available through the College of William & Mary Scholar Works (10.25773/hamz-zc50).
