# Supplementary material for: Seabed Resuspension in the Chesapeake Bay: Implications for Biogeochemical Cycling and Hypoxia
Source: Estuaries Coast. 2020 Jun 9;44(1):103–22. doi: 10.1007/s12237-020-00763-8 (PMC7752872; doi:10.1007/s12237-020-00763-8)
Supplement: Supplementary file 1 — (DOCX 67 kb) [file 12237_2020_763_MOESM1_ESM.docx]

**Supplement A: Statistics from Model-Observation Comparison for “Seabed Resuspension in the Chesapeake Bay: Implications for Biogeochemical Cycling and Hypoxia”**

# Moriarty, Julia M.^1,2^, Friedrichs, Marjorie A.M. ^1^, Harris, Courtney K. ^1^

^1^Virginia Institute of Marine Science, William & Mary, Gloucester Point, Virginia, 23062 USA

^2^Now at: Department of Atmospheric and Oceanic Sciences and Institute of Arctic and Alpine Research, University of Colorado Boulder, Boulder, CO, 80303 USA

Corresponding author: J. M. Moriarty, julia.moriarty@colorado.edu, Phone: (+1) 508-457-2306, Fax: 508-457-2310

# Abstract

This supplement provides quantitative comparisons between model results and observations, focusing on variability along the thalweg of Chesapeake Bay.

***Table A1:*** *Comparison of estimates from the Reference model run to observations from the Chesapeake Bay Program for salinity (in ppt). Each observation was compared to the estimate from the nearest model grid cell. RMSD was defined as the root mean squared difference. Statistics were computed for all regions of the Bay thalweg, including the Oligohaline Bay (OB), Upper Bay (UB), Mid Bay (MB), and Lower Bay (LB).*

| ***Variable*** | ***Year^*^*** | ***Mean*** | | ***2 Standard Errors*** | | ***Bias*** | ***RMSD*** | ***Unbiased RMSD*** | ***Number of Observations*** |
| --- | --- | --- | --- | --- | --- | --- | --- | --- | --- |
|  |  | ***Model*** | ***Observed*** | ***Model*** | ***Observed*** |  |  |  |  |
| ***OB, Surface*** | *2002* | *0.0* | *0.4* | *0.0* | *0.0* | *-0.4* | *0.4* | *0* | *14* |
|  | *2003* | *0.0* | *0.1* | *0.0* | *0.0* | *-0.1* | *0.1* | *0* | *29* |
| ***UB, Surface*** | *2002* | *9.1* | *8.4* | *0.2* | *0.4* | *0.6* | *1.3* | *1.1* | *150* |
|  | *2003* | *8.9* | *5.9* | *0.0* | *0.2* | *2.9* | *3.3* | *1.5* | *257* |
| ***MB, Surface*** | *2002* | *14.3* | *14.6* | *0.4* | *0.4* | *-0.2* | *0.6* | *0.5* | *133* |
|  | *2003* | *12.5* | *11.1* | *0.2* | *0.2* | *1.4* | *1.5* | *0.5* | *196* |
| ***LB, Surface*** | *2002* | *23.6* | *24.2* | *0.8* | *1.0* | *-0.7* | *1.1* | *0.9* | *66* |
|  | *2003* | *17.7* | *15.5* | *0.0* | *0.0* | *2.2* | *2.2* | *0.0* | *46* |
| ***OB, Bottom*** | *2002* | *0.0* | *0.5* | *0.0* | *0.0* | *-0.5* | *0.5* | *0.1* | *29* |
|  | *2003* | *0.0* | *0.1* | *0.0* | *0.0* | *-0.1* | *0.1* | *0.0* | *60* |
| ***UB, Bottom*** | *2002* | *18.1* | *19.7* | *0.2* | *0.3* | *-1.6* | *1.6* | *0.4* | *26* |
|  | *2003* | *17.6* | *17.9* | *0.1* | *0.3* | *-0.2* | *0.8* | *0.8* | *53* |
| ***MB, Bottom*** | *2002* | *20.6* | *19.9* | *0.3* | *0.3* | *0.7* | *1.9* | *1.8* | *431* |
|  | *2003* | *19.9* | *17.9* | *0.2* | *0.2* | *2.0* | *2.5* | *1.5* | *854* |
| ***LB, Bottom*** | *2002* | *27.2* | *27.1* | *0.2* | *0.3* | *0.0* | *1.3* | *1.3* | *289* |
|  | *2003* | *26.3* | *25.9* | *0.2* | *0.3* | *0.4* | *2.1* | *2.0* | *546* |

^*^Note that all model estimates are from over May 1 – July 31 for either 2002 or 2003.

***Table A2:*** *Same as Table A1, but for TSS (in mg L^-1^).*

| ***Variable*** | ***Year^*^*** | ***Mean*** | | ***2 Standard Errors*** | | ***Bias*** | ***RMSD*** | ***Unbiased RMSD*** | ***Number of Observations*** |
| --- | --- | --- | --- | --- | --- | --- | --- | --- | --- |
|  |  | ***Model*** | ***Observed*** | ***Model*** | ***Observed*** |  |  |  |  |
| ***OB, Surface*** | *2002* | *15.0* | *10.7* | *0.2* | *0.2* | *4.2* | *4.2* | *0.1* | *36* |
|  | *2003* | *12.8* | *15.4* | *0.3* | *0.5* | *-2.6* | *2.7* | *0.7* | *32* |
| ***UB, Surface*** | *2002* | *7.7* | *6.5* | *0.0* | *0.0* | *1.2* | *1.2* | *0.0* | *13* |
|  | *2003* | *7.3* | *6.9* | *0.0* | *0.0* | *0.4* | *0.4* | *0.0* | *10* |
| ***MB, Surface*** | *2002* | *4.1* | *5.9* | *0.25* | *0.15* | *-1.8* | *2.2* | *0.8* | *58* |
|  | *2003* | *5.8* | *7.2* | *0.1* | *0.2* | *-1.4* | *1.7* | *0.5* | *50* |
| ***LB, Surface*** | *2002* | *2.9* | *8.4* | *0.0* | *0.0* | *-5.6* | *5.6* | *0.0* | *13* |
|  | *2003* | *4.4* | *8.1* | *0.05* | *0.2* | *-3.7* | *3.8* | *0.4* | *38* |
| ***OB, Bottom*** | *2002* | *17.8* | *18.8* | *2.4* | *4.7* | *-1.0* | *4.7* | *4.7* | *17* |
|  | *2003* | *14.5* | *22.7* | *0.1* | *2.8* | *-8.2* | *9.1* | *4.3* | *10* |
| ***UB, Bottom*** | *2002* | *9.2* | *9.1* | *0.1* | *0.5* | *0.1* | *0.8* | *0.9* | *18* |
|  | *2003* | *9.3* | *10.7* | *0.0* | *0.0* | *-1.4* | *1.4* | *0.0* | *16* |
| ***MB, Bottom*** | *2002* | *3.9* | *11.4* | *0.2* | *2.0* | *-7.5* | *12.1* | *9.6* | *99* |
|  | *2003* | *4.0* | *9.7* | *0.2* | *1.0* | *-5.7* | *7.4* | *4.7* | *87* |
| ***LB, Bottom*** | *2002* | *5.2* | *13.6* | *0.5* | *0.7* | *-8.4* | *8.5* | *1.4* | *50* |
|  | *2003* | *5.2* | *11.6* | *0.3* | *0.8* | *-6.3* | *6.8* | *2.4* | *77* |

^*^Note that all model estimates are from over May 1 – July 31 for either 2002 or 2003.

***Table A3:*** *Same as Table A1, but for K_D_ (in m^-1^).*

| ***Variable*** | ***Year^*^*** | ***Mean*** | | ***2 Standard Errors*** | | ***Bias*** | ***RMSD*** | ***Unbiased RMSD*** | ***Number of Observations*** |
| --- | --- | --- | --- | --- | --- | --- | --- | --- | --- |
|  |  | ***Model*** | ***Observed*** | ***Model*** | ***Observed*** |  |  |  |  |
| ***OB, Surface*** | *2002* | *2.4* | *1.6* | *0.1* | *0.1* | *0.7* | *0.8* | *0.3* | *20* |
|  | *2003* | *2.4* | *2.4* | *0.1* | *0.3* | *0.0* | *0.6* | *0.6* | *15* |
| ***UB, Surface*** | *2002* | *1.4* | *1.2* | *0.1* | *0.1* | *0.3* | *0.4* | *0.3* | *20* |
|  | *2003* | *1.5* | *1.5* | *0.1* | *0.3* | *-0.1* | *0.5* | *0.5* | *13* |
| ***MB, Surface*** | *2002* | *0.8* | *0.7* | *0.1* | *0.1* | *0.1* | *2.2* | *0.8* | *58* |
|  | *2003* | *1.0* | *1.0* | *0.0* | *0.1* | *0.0* | *0.3* | *0.3* | *33* |
| ***LB, Surface*** | *2002* | *0.3* | *0.6* | *0.1* | *0.1* | *-0.3* | *0.4* | *0.3* | *25* |
|  | *2003* | *0.6* | *0.8* | *0.1* | *0.1* | *-0.2* | *0.3* | *0.2* | *23* |

^*^Note that all model estimates are from over May 1 – July 31 for either 2002 or 2003.

***Table A4:*** *Same as Table A1, but for O_2_ (in mg O_2_ L^-1^).*

| ***Variable*** | ***Year^*^*** | ***Mean*** | | ***2 Standard Errors*** | | ***Bias*** | ***RMSD*** | ***Unbiased RMSD*** | ***Number of Observations*** |
| --- | --- | --- | --- | --- | --- | --- | --- | --- | --- |
|  |  | ***Model*** | ***Observed*** | ***Model*** | ***Observed*** |  |  |  |  |
| ***OB, Surface*** | *2002* | *7.9* | *7.5* | *0.0* | *0.0* | *0.4* | *0.4* | *0.0* | *14* |
|  | *2003* | *7.1* | *7.9* | *0.0* | *0.0* | *-0.8* | *0.8* | *0.0* | *15* |
| ***UB, Surface*** | *2002* | *7.7* | *6.3* | *0.0* | *0.0* | *1.1* | *0.0* | *1.1* | *81* |
|  | *2003* | *7.8* | *6.9* | *0.1* | *0.1* | *1.0* | *1.0* | *0.4* | *138* |
| ***MB, Surface*** | *2002* | *9.3* | *8.1* | *0.1* | *0.1* | *1.2* | *1.3* | *0.4* | *85* |
|  | *2003* | *10.4* | *8.3* | *0.1* | *0.1* | *2.2* | *2.4* | *0.6* | *126* |
| ***LB, Surface*** | *2002* | *8.3* | *8.5* | *0.0* | *0.0* | *-0.2* | *1.1* | *1.0* | *26* |
|  | *2003* | *9.5* | *8.4* | *0.1* | *0.1* | *1.1* | *1.1* | *0.0* | *27* |
| ***OB, Bottom*** | *2002* | *7.9* | *7.3* | *0.0* | *0.1* | *0.6* | *0.6* | *0.2* | *29* |
|  | *2003* | *7.1* | *7.9* | *0.0* | *0.0* | *-0.8* | *0.8* | *0.0* | *31* |
| ***UB, Bottom*** | *2002* | *2.6* | *0.6* | *0.2* | *0.0* | *1.9* | *1.9* | *0.1* | *26* |
|  | *2003* | *--* | *--* | *--* | *--* | *--* | *--* | *--* | *0* |
| ***MB, Bottom*** | *2002* | *5.9* | *3.1* | *0.1* | *0.2* | *2.8* | *3.0* | *0.9* | *443* |
|  | *2003* | *3.6* | *2.1* | *0.2* | *0.2* | *1.5* | *1.8* | *1.0* | *440* |
| ***LB, Bottom*** | *2002* | *8.8* | *6.7* | *0.1* | *0.1* | *2.0* | *2.1* | *0.6* | *289* |
|  | *2003* | *8.7* | *6.8* | *0.2* | *0.2* | *1.8* | *2.0* | *0.9* | *274* |

^*^Note that all model estimates are from over May 1 – July 31 for either 2002 or 2003.

***Table A5:*** *Same as Table A1, but for NH_4_ (in mg N L^-1^).*

| ***Variable*** | ***Year^*^*** | ***Mean*** | | ***2 Standard Errors*** | | ***Bias*** | ***RMSD*** | ***Unbiased RMSD*** | ***Number of Observations*** |
| --- | --- | --- | --- | --- | --- | --- | --- | --- | --- |
|  |  | ***Model*** | ***Observed*** | ***Model*** | ***Observed*** |  |  |  |  |
| ***OB, Surface*** | *2002* | *0.12* | *0.08* | *0.00* | *0.00* | *0.04* | *0.04* | *0.01* | *37* |
|  | *2003* | *0.13* | *0.08* | *0.01* | *0.01* | *0.05* | *0.07* | *0.01* | *27* |
| ***UB, Surface*** | *2002* | *0.02* | *0.02* | *0.00* | *0.00* | *-0.00* | *0.00* | *0.00* | *13* |
|  | *2003* | *0.03* | *0.06* | *0.00* | *0.00* | *-0.03* | *0.03* | *0.0* | *10* |
| ***MB, Surface*** | *2002* | *0.01* | *0.01* | *0.00* | *0.00* | *-0.01* | *0.01* | *0.00* | *58* |
|  | *2003* | *0.01* | *0.04* | *0.00* | *0.00* | *-0.03* | *0.03* | *0.01* | *50* |
| ***LB, Surface*** | *2002* | *0.00* | *0.01* | *0.00* | *0.00* | *-0.01* | *0.01* | *0.00* | *42* |
|  | *2003* | *0.01* | *0.03* | *0.00* | *0.00* | *-0.02* | *0.02* | *0.01* | *38* |
| ***OB, Bottom*** | *2002* | *0.16* | *0.10* | *0.01* | *0.01* | *0.06* | *0.06* | *0.01* | *17* |
|  | *2003* | *0.2* | *0.1* | *0.01* | *0.05* | *0.10* | *0.13* | *0.08* | *17* |
| ***UB, Bottom*** | *2002* | *0.19* | *0.21* | *0.00* | *0.00* | *-0.02* | *0.02* | *0.00* | *18* |
|  | *2003* | *0.23* | *0.43* | *0.00* | *0.00* | *-0.20* | *0.20* | *0.00* | *16* |
| ***MB, Bottom*** | *2002* | *0.10* | *0.06* | *0.01* | *0.01* | *0.04* | *0.05* | *0.03* | *108* |
|  | *2003* | *0.20* | *0.24* | *0.01* | *0.03* | *-0.05* | *0.11* | *0.10* | *87* |
| ***LB, Bottom*** | *2002* | *0.02* | *0.02* | *0.01* | *0.0* | *-0.01* | *0.01* | *0.01* | *79* |
|  | *2003* | *0.07* | *0.06* | *0.01* | *0.01* | *0.01* | *0.03* | *0.02* | *77* |

^*^Note that all model estimates are from over May 1 – July 31 for either 2002 or 2003.

***Table A6:*** *Same as Table A1, but for NO_2+3_ (in mg N L^-1^).*

| ***Variable*** | ***Year^*^*** | ***Mean*** | | ***2 Standard Errors*** | | ***Bias*** | ***RMSD*** | ***Unbiased RMSD*** | ***Number of Observations*** |
| --- | --- | --- | --- | --- | --- | --- | --- | --- | --- |
|  |  | ***Model*** | ***Observed*** | ***Model*** | ***Observed*** |  |  |  |  |
| ***OB, Surface*** | *2002* | *0.61* | *0.48* | *0.05* | *0.08* | *0.13* | *0.13* | *0.05* | *32* |
|  | *2003* | *0.69* | *0.69* | *0.03* | *0.02* | *-0.00* | *0.04* | *0.04* | *60* |
| ***UB, Surface*** | *2002* | *0.19* | *0.22* | *0.00* | *0.00* | *-0.03* | *0.03* | *0.00* | *13* |
|  | *2003* | *0.31* | *0.33* | *0.00* | *0.00* | *-0.01* | *0.01* | *0.00* | *22* |
| ***MB, Surface*** | *2002* | *0.01* | *0.03* | *0.00* | *0.01* | *-0.02* | *0.03* | *0.02* | *59* |
|  | *2003* | *0.03* | *0.16* | *0.01* | *0.01* | *-0.13* | *0.13* | *0.03* | *107* |
| ***LB, Surface*** | *2002* | *0.00* | *0.00* | *0.00* | *0.00* | *0.00* | *0.00* | *0.00* | *40* |
|  | *2003* | *0.00* | *0.02* | *0.00* | *0.01* | *-0.02* | *0.03* | *0.02* | *79* |
| ***OB, Bottom*** | *2002* | *0.87* | *0.61* | *0.03* | *0.03* | *0.26* | *0.26* | *0.02* | *17* |
|  | *2003* | *0.95* | *0.83* | *0.01* | *0.02* | *0.13* | *0.13* | *0.03* | *36* |
| ***UB, Bottom*** | *2002* | *0.16* | *0.01* | *0.01* | *0.00* | *0.13* | *0.13* | *0.01* | *18* |
|  | *2003* | *0.28* | *0.09* | *0.03* | *0.02* | *0.20* | *0.20* | *0.01* | *47* |
| ***MB, Bottom*** | *2002* | *0.04* | *0.03* | *0.06* | *0.00* | *0.02* | *0.04* | *0.03* | *111* |
|  | *2003* | *0.10* | *0.08* | *0.01* | *0.01* | *0.02* | *0.06* | *0.06* | *181* |
| ***LB, Bottom*** | *2002* | *0.00* | *0.00* | *0.00* | *0.00* | *-0.00* | *0.00* | *0.00* | *74* |
|  | *2003* | *0.01* | *0.01* | *0.00* | *0.00* | *-0.00* | *0.00* | *0.00* | *158* |

^*^Note that all model estimates are from over May 1 – July 31 for either 2002 or 2003.

***Table A7:*** *Comparison of estimates from the Reference model run to those derived from summertime observations of primary productivity and oxygen consumption.*

| **Variable** | | **Peak Value** | | **Location of Along-estuary Maximum****** | | **Time Period and Citation for Observations** |
| --- | --- | --- | --- | --- | --- | --- |
|  |  | ***Modeled^**^*** | ***Observed^***^*** | ***Modeled*** | ***Observed*** |  |
| Primary Productivity^*^ | | 0.44 ± 0.08 mg C L^-1^ d^-1^ | 0.39 ± 0.02 mg C L^-1^ d^-1^ | ~38.9 ^o^N  (102 km) | ~38.4 – 38.75 ^o^N (150-200 km) | June-August;  Harding et al. (2002) |
| Bottom Water Oxygen Consumption | Lower Bay Peak | 0.01± 0.0003 mg O_2_ L^-1^ h^-1^ | ~0.01 - 0.04 mg O_2_ L^-1^ h^-1^ | 37.0 – 37.8 ^o^N (271-387 km) | 37.3 ^o^N  (341 km) | May-July;  Smith and Kemp (1995) |
|  | Oligohaline Bay Peak | 0.02± 0.002 mg O_2_ L^-1^ h^-1^ | ~0.005 – 0.015 mg O_2_ L^-1^ h^-1^ | 39.2 – 39.4 ^o^N (0-46 km) | 39.3 ^o^N  (26 km) |  |

^*^Values were estimated for the euphotic zone, which was assumed to be the surface 6 m of the water column based on estimates from Harding et al. (2002)’s Region 4.

^**^For model estimates, the location of the along-thalweg maximum was estimated (e.g. see Figure 5), then the average and standard deviation of values in that region were determined.

^***^Observational averages and standard deviations were based on all data from a given region (i.e. Region 4 from Harding et al., 2002), or a range of values were given for an individual station (i.e. Smith and Kemp, 1995)

^****^ Along-estuary locations are given in decimal degrees, and distance along the thalweg, as shown in Figure 1.
