# Supplementary material for: Seabed Resuspension in the Chesapeake Bay: Implications for Biogeochemical Cycling and Hypoxia
Source: Estuaries Coast. 2020 Jun 9;44(1):103–22. doi: 10.1007/s12237-020-00763-8 (PMC7752872; doi:10.1007/s12237-020-00763-8)
Supplement: Supplementary file 2 — (DOCX 400 kb) [file 12237_2020_763_MOESM2_ESM.docx]

**Supplement B: Supplemental Model Results from 2002 for “Seabed Resuspension in the Chesapeake Bay: Implications for Biogeochemical Cycling and Hypoxia”**

# Moriarty, Julia M.^1,2^, Friedrichs, Marjorie A.M. ^1^, Harris, Courtney K. ^1^

^1^Virginia Institute of Marine Science, William & Mary, Gloucester Point, Virginia, 23062 USA

^2^Now at: Department of Atmospheric and Oceanic Sciences and Institute of Arctic and Alpine Research, University of Colorado Boulder, Boulder, CO, 80303 USA

Corresponding author: J. M. Moriarty, julia.moriarty@colorado.edu, Phone: (+1) 508-457-2306, Fax: 508-457-2310

# Abstract

This supplement provides additional model analyses for 2002.

***
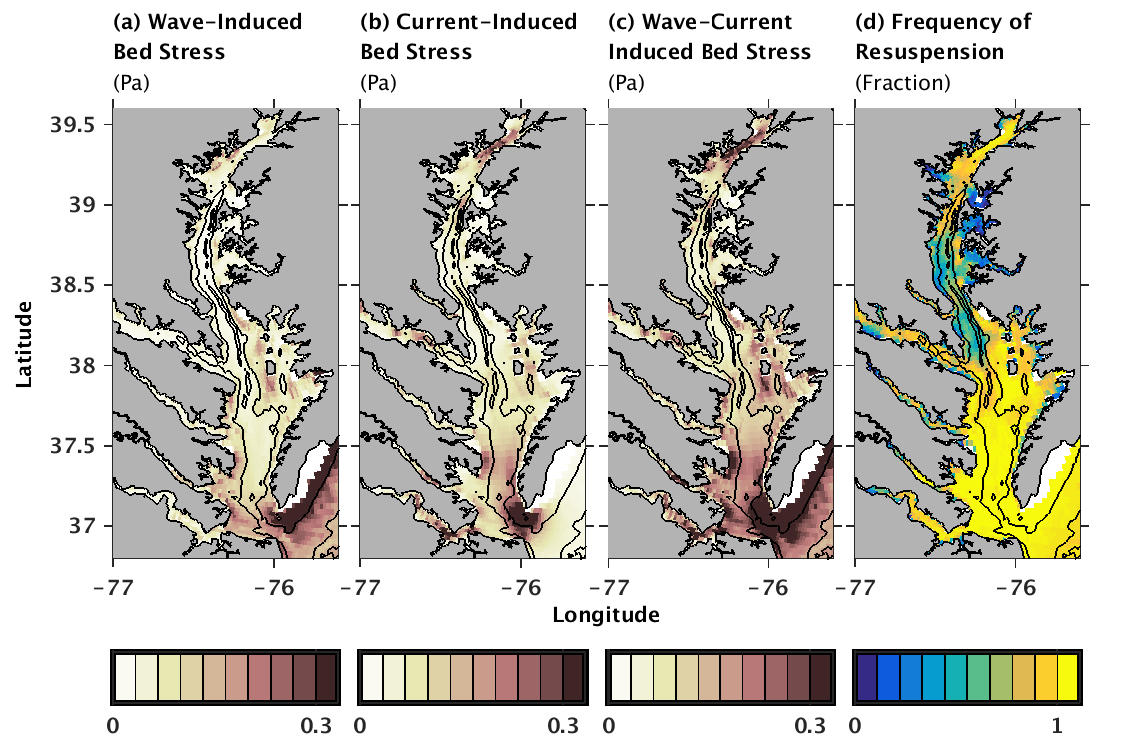
 Figure B1:*** *Same as Figure 4, but for 2002.*


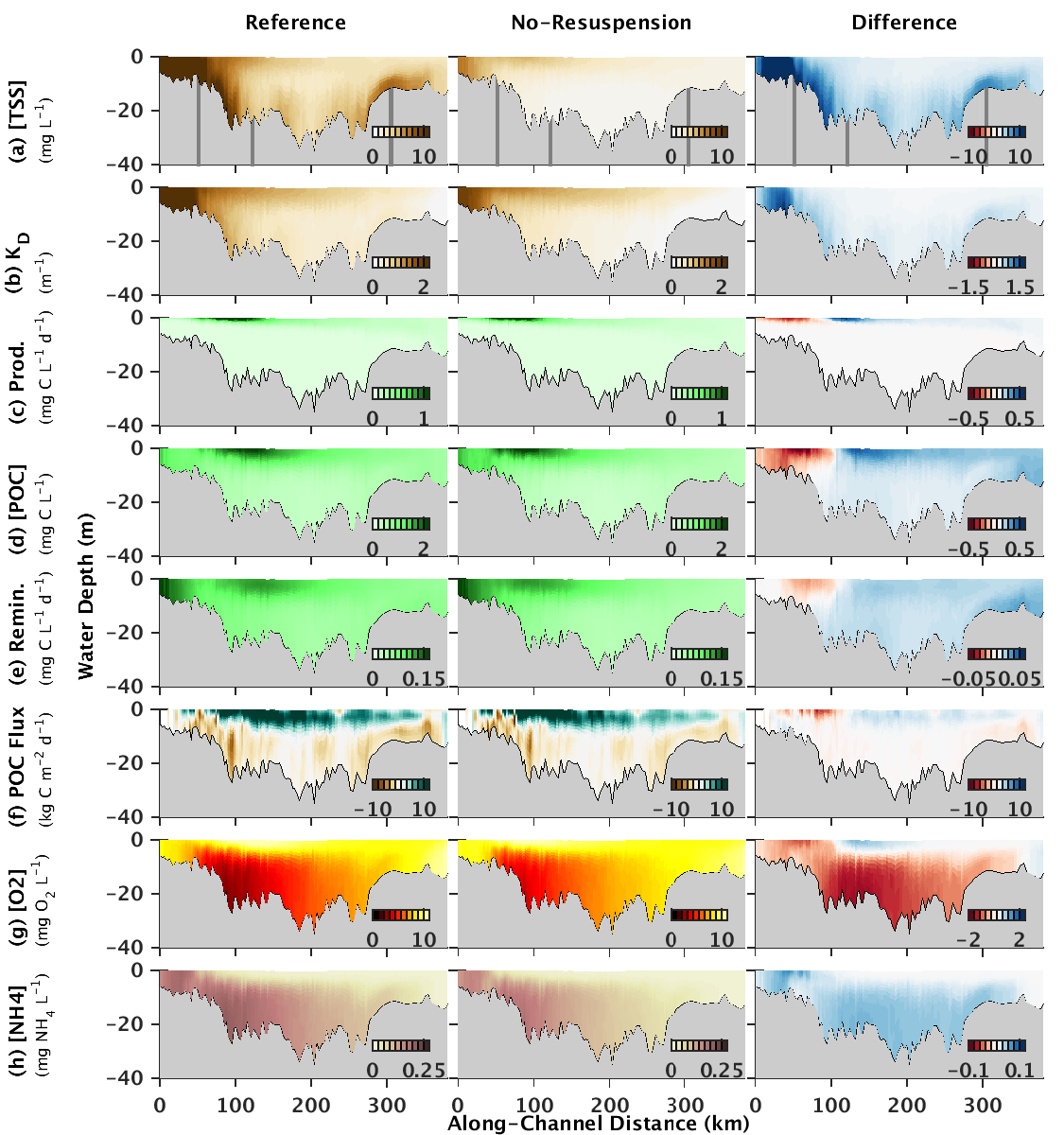


***Figure B2:*** *Same as Figure 5, but for 2002.*
